# From pixels to conservation: Deep learning for automated monitoring of rare and endangered wildlife

**DOI:** 10.1016/j.isci.2026.116669

**Published:** 2026-09-10

**Authors:** Donghua Xuan, Yuchen Cai, Minxi Xu, Chao Long, Nian Cai

**Affiliations:** 1 School of Information Engineering, Guangdong University of Technology, Guangzhou, China; 2 School of Engineering, Nanfang College Guangzhou, Guangzhou, China

**Keywords:** deep learning, wildlife monitoring, computer vision, species recognition, individual identification, conservation technology, camera traps

## Abstract

Conservation of rare and endangered species requires scalable monitoring solutions to address accelerating biodiversity loss. Deep learning has transformed wildlife monitoring, enabling automated species identification, individual tracking, demographic inference, and behavioral analysis from camera traps, drones, and video surveillance. This review systematically analyzes 159 wildlife monitoring studies (2014–2026) across four technical domains: species recognition, individual re-identification, soft biometrics (age/sex estimation), and behavior/pose analysis. We examine architectural evolution across three architectural paradigms—CNN-based models, Attention & Sequence Architectures, and Foundation Models- documenting performance advances and ecological applications. Key challenges—environmental interference, data scarcity, domain shift, computational constraints—and optimization strategies, including data augmentation, transfer learning, and model compression, are systematically addressed. Case studies demonstrate real-world impact across diverse taxa: giant pandas, Amur tigers, great apes, and marine mammals. We identify critical research gaps and emerging opportunities in foundation model adaptation, multimodal learning, spatiotemporal modeling, and edge deployment. This review bridges computer vision research and conservation practice, providing actionable insights for developing robust, deployable monitoring systems that translate algorithmic advances into conservation impact.

## Highlights

• 159 deep learning studies on CITES/IUCN-listed wildlife systematically reviewed
• Four domains: species recognition, re-ID, soft biometrics, and behavior analysis
• Architectural evolution: CNN-based, Attention & Sequence, and Foundation Models
• Six deployment challenges mapped to DL solutions with a forward-looking roadmap

## Graphical abstract

**Figure:**
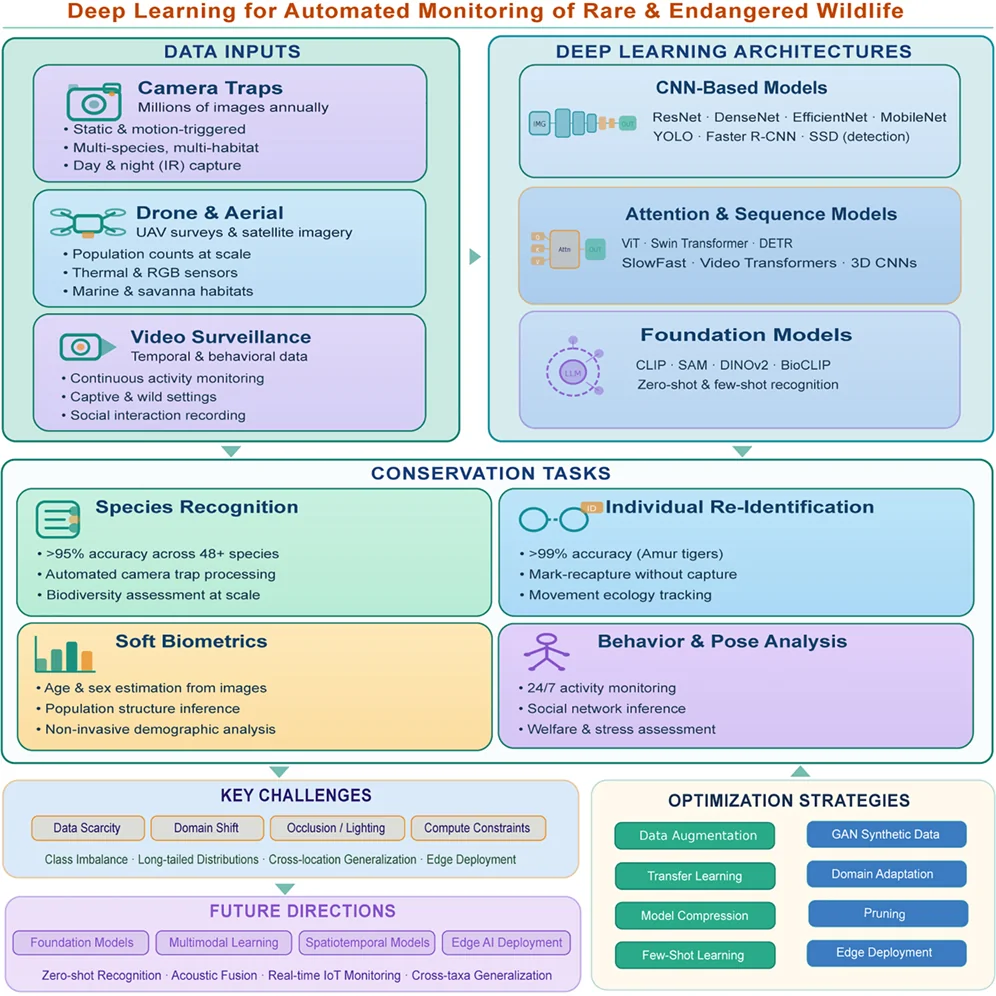

## Teaser

Environmental monitoring; Nature conservation; Zoology

## Introduction

### The conservation monitoring crisis

Global biodiversity faces unprecedented threats, with modern vertebrate extinction rates estimated to be up to 100 times higher than natural background levels.^1^ The crisis is widespread; research on the ongoing sixth mass extinction reveals that the human-driven extinction event is more severe than previously assessed and is rapidly accelerating, with generic extinction rates already 35 times higher than background levels.^2^ Effective conservation requires timely, accurate data on species distributions, population dynamics, and behaviors to guide interventions. Traditional monitoring—point surveys, line transects, mark-recapture—provides critical baseline data but faces fundamental limitations: high costs, labor intensity, observer bias, limited spatial-temporal coverage, and wildlife disturbance. Camera trap networks now generate millions of images annually worldwide, yet analysis capacity lags catastrophically. In 2023, Wolong National Nature Reserve’s 500,000 giant panda images required an estimated year of expert labor for manual processing.^3^ This bottleneck is global, creating vast repositories of unanalyzed conservation data precisely when rapid intervention decisions are most critical.

**Figure 1:**
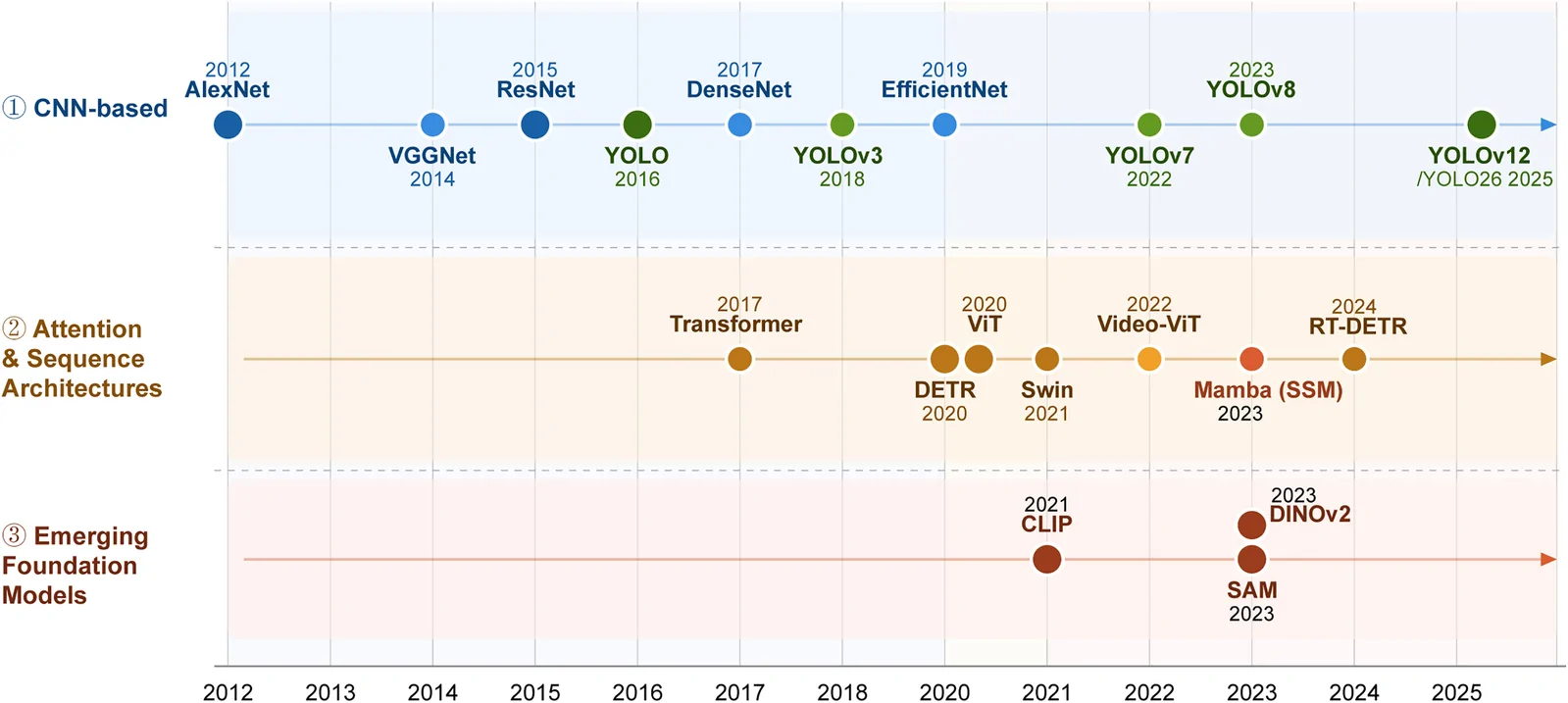
Three paradigm shifts in deep learning architectures for wildlife monitoring (2012–2025) Era boundaries reflect dominant innovation waves, not hard cutoffs. YOLO-series detectors (CNN backbones) are classified under ①; DETR/RT-DETR (Transformer encoder-decoder) under ②; Mamba (SSM) is placed in ② as a linear-complexity alternative to self-attention. Earlier architectures remain in active use and are frequently combined with later paradigms.

The emergence of “conservation technology” as a distinct interdisciplinary field has created new opportunities for scalable biodiversity monitoring.^4^ Deep learning offers transformative solutions: since AlexNet’s 2012 breakthrough,^5^ three successive architectural paradigms have redefined what automated wildlife monitoring can achieve (Figure 1). Convolutional Neural Networks (CNNs) established species recognition as a tractable problem; Attention-based architectures and video Transformers extended this to temporal behavior analysis; and Foundation Models (large-scale pre-trained models emerging since 2021) now enable zero-shot and few-shot recognition across taxa for which no labeled training data exist. Rapid architectural evolution—from early CNNs through YOLO (You Only Look Once) detectors^6^ to Vision Transformers^7^ —has continuously expanded capabilities, transforming the bottleneck of unanalyzed imagery into a tractable computational pipeline.

**Figure 2:**
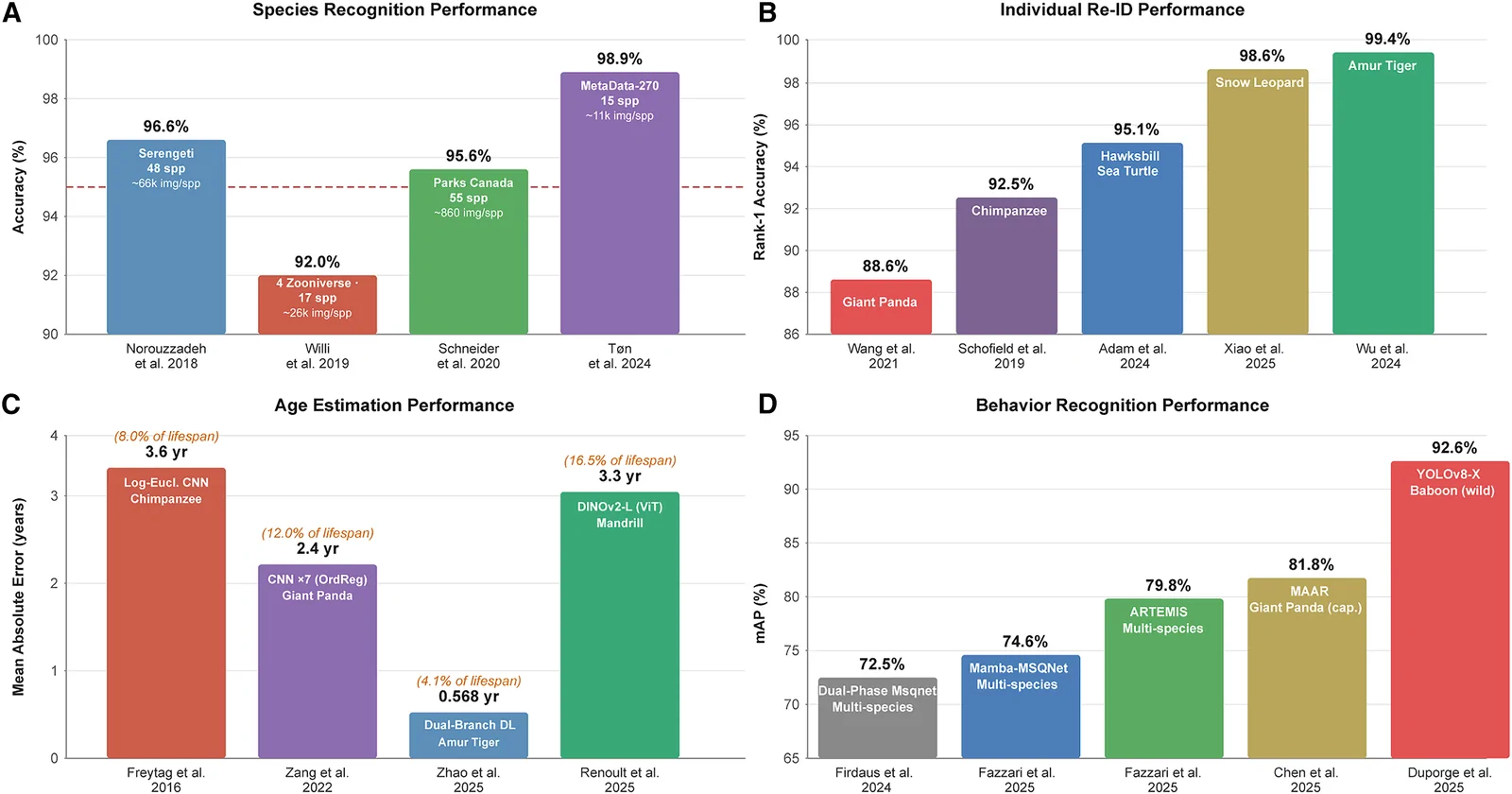
Performance benchmarks across four technical domains of wildlife deep learning (A) Species recognition accuracy for four camera-trap datasets (sorted by year); bar labels show accuracy (%), dataset name, species count, and mean training images per species (img/spp); dashed red line = 95% threshold. (B) Individual re-identification Rank-1 accuracy (proportion of queries where the correct individual ranks first) for five species: Giant Panda (Wang et al.^12^), Chimpanzee (Schofield et al.^13^), Hawksbill Sea Turtle (Adam et al.^15^), Snow Leopard (Xiao et al.^16^), and Amur Tiger (Wu et al.^14^). (C) Age estimation mean absolute error (MAE, years) for four studies; orange italic values indicate MAE as a percentage of mean species lifespan (Chimpanzee 45 years, Giant Panda 20 years, Mandrill 20 years, Amur Tiger 14 years); Log-Eucl. CNN = Log-Euclidean CNN; OrdReg = Ordinal Regression; DINOv2-L = DINOv2-Large (ViT); Dual-Branch DL = Dual-Branch Deep Learning. (D) Behavior recognition mean average precision (mAP) for five wildlife-applied deep learning models (sorted by mAP); bar labels show model architecture and study organism; Dual-Phase Msqnet = Dual-Phase Multi-Scale Queue Network for species-specific activity recognition (Yu et al.^21^); Mamba-MSQNet = Mamba-based Multi-Scale Queue Network for multi-label animal action recognition (Fazzari et al.^22^); ARTEMIS = Animal Recognition Through Enhanced Multimodal Integration System for multi-label behavior recognition (Fazzari et al.^24^); MAAR = Multi-Attentional Action Recognition method for captive giant panda surveillance video (Duan et al.^23^); YOLOv8-X = YOLOv8-X applied to drone video behavior detection in wild baboons (Duporge et al.^25^).

Modern systems now enable four categories of wildlife insight: (1) automated species recognition with accuracy exceeding 93% (e.g., 96.6% on 48 species^8^), advancing the vision of integrative taxonomy through machine learning^9^^,^^10^^,^^11^; (2) individual re-identification exploiting natural markings (e.g., coat patterns, fin contours, spot arrangements) to enable non-invasive population census^12^^,^^13^^,^^14^^,^^15^^,^^16^; (3) demographic inference through soft biometric estimation (e.g., age class, body condition, sex)^17^^,^^18^^,^^19^^,^^20^; and (4) behavioral analysis via spatiotemporal video understanding.^21^^,^^22^^,^^23^^,^^24^^,^^25^ These capabilities, benchmarked across species and architectures (Figures 2A–2D), demonstrate that deep learning now facilitates continuous, non-invasive monitoring at scales previously unimaginable, enabling data-driven conservation management.^26^^,^^27^

### Current state of knowledge

Five previous studies have reviewed machine learning applications in wildlife monitoring. Table 1 provides a comparative analysis of wildlife monitoring reviews involving five previous studies and our review, in which these reviews are statistically analyzed in terms of three parts, such as Scope & Positioning, Technical Domains, and Unique Contributions.

**Table 1:** Comparative analysis of wildlife monitoring reviews: This review against five previous works

For Scope & Positioning, we analyze these review works in four dimensions, including publication type & article count, target species scope, primary data source, and deep learning era coverage. Specifically, five previous works are perspective or narrative reviews lacking a formally reproducible search protocol. Comparatively, our review follows a systematic methodology compliant with STAR Methods, yielding 159 primary wildlife deep learning studies from 159 total references. It is the only work to focus exclusively on CITES-listed and IUCN Red List threatened species, to integrate camera traps, UAVs, and multi-angle video in a unified framework, and to trace the full architectural evolution across three paradigms: CNN-based models (2015–present), Attention & Sequence Architectures (2017–present), and Foundation Models (2021–present). By contrast, Kumar and Singh^28^ and Ravoor and Sudarshan^29^ are confined to pre-deep-learning or early CNN-only periods, and Vogg et al.^30^ is restricted to primates.

Four technical domains are also analyzed for these review works, including species recognition & biodiversity assessment, individual re-identification, soft biometrics (age/sex/condition estimation), behavior analysis & pose estimation. For species recognition and biodiversity assessment, Kumar and Singh^28^ relied exclusively on pre-deep-learning biometric features, missing the CNN-to-Attention & Sequence Architecture trajectory that now underpins multi-species recognition across 48+ species at >96% accuracy. Ravoor and Sudarshan^29^ covered early CNN approaches but predated Vision Transformers and Foundation Models entirely. For individual re-identification, Schneider et al.^26^ provided a narrowly scoped survey of early CNN re-ID restricted to camera trap imagery, without addressing triplet loss, part-based metric learning, or cross-species generalization that now enable 99.37% accuracy for Amur tiger re-identification. For soft biometrics (age, sex, and condition estimation), no previous review work treats this domain systematically. Tuia et al.^31^ mentioned age and sex only as ecological variables without technical analysis, while all other reviewed works omit the domain entirely. For behavior analysis and pose estimation, Vogg et al.^30^ provided the most recent and technically detailed treatment, but restricted exclusively to primates. Also, it does not deal with multi-taxon 2D/3D pose estimation, Video Transformer architectures, and behavioral ecology integration.

For Unique Contributions, we analyze these review works in the dimensions of challenge, solution framework, practical deployment & edge AI, conservation impact evidence, and future research roadmap. Among five previous review works, only one work covers these four dimensions. Moreover, no previous review work provides: (1) an integrated analysis spanning all four technical domains within a unified conservation-focused framework; (2) a systematic challenge-to-solution mapping linking real-world deployment obstacles—data scarcity, domain shift, class imbalance, and computational constraints—to validate deep learning optimization strategies; (3) comprehensive coverage of the full architectural evolution across three paradigms: CNN-based models (2015–present), Attention & Sequence Architectures (2017–present), and Foundation Models (2021–present).

### Scope and contributions

This review addresses the three above issues existing in five previous review works through systematic analysis of deep learning applications for CITES-listed and IUCN (International Union for Conservation of Nature) Red List species from 2014 to 2026. The contributions of this review can be summarized as follows.

• Four-domain technical synthesis. We categorize existing deep learning-based studies for wildlife conservation into four technical domains, which are (1) species recognition and biodiversity assessment (CNN-based/Attn & Seq/FM architectures, multi-ecosystem benchmarks); (2) individual re-identification (deep metric learning, triplet loss, part-based models, 99.37% accuracy for Amur tigers^14^). (3) soft biometrics—age, sex, and condition estimation (ordinal regression, multi-task CNNs, population structure inference); and (4) behavior analysis and pose estimation (Video Transformers, 2D/3D keypoint detection, behavioral ecology integration).
• Full architectural trajectory with conservation focus. Existing deep learning based methods for wildlife conservation are categorized in terms of network architecture, involving (1) CNN-based Models (2015–present), encompassing classification backbones (ResNet, DenseNet, EfficientNet) and convolutional detection pipelines (YOLO series, Faster R-CNN, RetinaNet); (2) Attention & Sequence Architectures (2020–present), including Vision Transformers (ViT, Swin), Video Transformers, Mamba models; and (3) Foundation Models (2021–present; CLIP, SAM, DINO), with performance trajectories documented specifically for CITES-listed and IUCN Red List species—a scope absent from all previous reviews.
• Challenge-to-solution framework with deployment roadmap. A structured mapping is provided to potential solutions to six real-world deployment obstacles—environmental interference, data scarcity, class imbalance, domain shift, individual variation, and computational constraints. Also, a forward-looking roadmap is provided across four emerging research themes: Foundation Models, multimodal sensor fusion, spatiotemporal modeling, and sustainable edge AI.

The remainder of this review is structured as follows. Results section provides the statistical analyses of existing AI studies for wildlife monitoring, in which full architectural trajectory with a conservation focus is summarized in the Evolution of Deep Learning Architectures for Wildlife Applications subsection and four-domain technical synthesis is discussed in four subsections in details, including the subsections of Species Recognition and Biodiversity Assessment, Individual Re-Identification Systems, Demographic Analysis via Soft Biometrics, and Behavioral Understanding and Pose Estimation. Then, a challenge-to-solution framework with a deployment roadmap is provided in the Discussion section. Next, some conclusions are provided in the Conclusions section. Subsequently, the detailed methodology is described in STAR Methods section. Finally, some other information is provided after STAR Methods section, such as supplement information, abbreviations, acknowledgments, author contributions, declaration of interests, and references.

## Results

### Overview of deep learning in wildlife monitoring

#### Evolution of deep learning architectures for wildlife applications

The application of deep learning to wildlife monitoring has progressed through distinct architectural eras, each expanding capabilities while addressing prior limitations. This evolution parallels broader computer vision advances but with wildlife-specific adaptations addressing unique challenges: limited training data for rare species, extreme class imbalance, environmental variability, and computational constraints for field deployment.

##### CNN-based models (2015–present)

AlexNet^5^ established deep learning feasibility, demonstrating that deep CNNs could learn hierarchical visual representations from raw pixels. Early wildlife applications adapted established CNN architectures (VGGNet, GoogLeNet), achieving promising but inconsistent results due to limited training data. ResNet^32^ enabled substantially deeper networks through residual connections, dramatically improving feature learning capacity. Norouzzadeh et al.^8^ demonstrated ResNet-152’s transformative potential, achieving 96.6% accuracy on 3.2 million Snapshot Serengeti images across 48 species, matching human expert performance while processing in seconds what required months of manual effort. DenseNet improved feature reuse through dense connectivity, beneficial for fine-grained species discrimination.

Real-world camera traps require full-scene localization; CNN-based detection pipelines address this need. Two-stage detectors such as Faster R-CNN (Region-based Convolutional Neural Network)^33^ provided high precision but limited speed. YOLO revolutionized real-time detection through a single-stage architecture, enabling video stream processing. Successive YOLO iterations progressively improved accuracy-speed tradeoffs through architectural innovations—including multi-scale feature fusion, bag-of-freebies training strategies, and re-parameterization techniques^34^ —making real-time wildlife detection practical. Wei et al.^35^ demonstrated wildlife-adapted YOLO achieving 93.8% mAP (mean Average Precision) at 30+ FPS (frames per second), enabling real-time human-wildlife conflict mitigation. RetinaNet^36^ addressed extreme class imbalance through focal loss, particularly valuable for rare species comprising <1% of camera trap images.

##### Attention & sequence architectures (2020–present)

This category encompasses Vision Transformers (ViT, Swin Transformer), Video Transformers for temporal modeling, and hybrid architectures combining CNN inductive biases with self-attention mechanisms, as well as state-space sequence models such as Mamba^22^^,^^37^ (and its hybrid variant MambaVision) when used independently of the foundation model paradigm. Transformer architectures,^38^ initially developed for natural language processing, demonstrated surprising effectiveness for vision when adapted as Vision Transformers (ViT). Unlike CNNs’ hierarchical local feature learning, Transformers model global context through self-attention, capturing long-range dependencies crucial for understanding animals in complex natural scenes. Swin Transformer^39^ introduced a hierarchical architecture and shifted windows, achieving state-of-the-art performance while reducing computational costs. For wildlife monitoring, Transformers excel at: (1) relating animals to distant environmental context (e.g., prey-predator spatial relationships), (2) temporal modeling across video frames for behavior analysis,^3^^,^^40^^,^^41^^,^^42^ and (3) handling severe occlusion by attending to visible body parts. Attention mechanisms also enable model interpretability through visualization of salient image regions, building trust for conservation decision-making. However, Transformers typically require larger training datasets than CNNs, challenging for rare species. Hybrid architectures combining CNN inductive biases with Transformer global modeling show promise for data-efficient wildlife applications.

##### Foundation models (2021–present)

Large-scale pre-training on massive datasets has produced “foundation models” with remarkable zero-shot and few-shot capabilities to solve a core problem in wildlife monitoring: recognizing species or behaviors for which no annotated training images exist. Specifically, CLIP^40^ learns joint vision-language representations to enable open-vocabulary recognition, while MSQNet^43^ extends this to classify 140+ behavioral categories across 850 species. SAM (Segment Anything Model)^41^ addresses a complementary challenge of locating and delineating individual animals in cluttered field imagery without species-specific training; it is trained on 1.1 billion masks and can be generalized to unseen taxa. Allabadi et al.^44^ demonstrated 56.2%–61.6% AP across four unseen mammal species in open-world monitoring. DINOv2^20^^,^^45^ addresses another problem: how to recognize the same individual animal across repeated encounters without invasive marking. Through self-supervised Vision Transformer pre-training, DINOv2 produces transferable dense visual features with no text or label supervision for age estimation in wild mandrills^20^ and individual re-identification of western lowland gorillas.^45^ DINO-X,^46^ an extension employing open-vocabulary grounding, further enables detection of previously unseen species without task-specific retraining. Generally, CLIP identifies species and behaviors, and SAM delineates individuals in complex scenes. DINO re-identifies individuals across encounter, enabling end-to-end population monitoring without species-specific training.

For clear clarification, these deep learning-based wildlife monitoring methods are statistically analyzed for five taxonomic classes with nine taxonomic orders, shown in Table 2. As indicated in Table 2, 79.8% of 159 related studies deal with Mammalia, with five orders of Carnivora (32.7%), Primates (19.5%), Artiodactyla (11.3%), Cetacea & Pinnipedia (8.8%), and Proboscidea (7.5%). The remaining four taxonomic classes involve Aves (10.1%), Reptilia (5.0%), Actinopterygii (3.1%), and Insecta & Invertebrata (1.9%). This mammal-heavy distribution reflects benchmark dataset availability and conservation funding patterns rather than actual biodiversity, a structural bias discussed further in the Dataset limitations section.

**Table 2:** Taxonomic composition of reviewed studies and deep learning architecture distribution across animal groups

**Note:** n = number of studies (2014–2026, *n* = 159 total). Architecture percentages are non-exclusive (one study may use multiple architecture types). consequently, column percentages within a taxonomic group may sum to >100%. IUCN status based on IUCN Red List (2022–2024); CR = Critically Endangered; EN = Endangered; VU = Vulnerable; NT = Near Threatened. Architecture categories are defined as follows: CNN-based Models includes all CNN backbone architectures (ResNet, VGG, Inception, MobileNet, EfficientNet), all YOLO-series detectors (YOLOv3–v12, YOLOX), and Faster R-CNN; Attn. & Seq. Arch. includes Vision Transformer (ViT), Swin Transformer, Video Swin, VideoMAE, TimeSformer, DETR and its variants (RT-DETR, DINO-detection), and Mamba/SSM sequence models; FM (Foundation Models) includes CLIP,^40^ SAM,^41^ and DINO/DINOv2,^20^^,^^45^ deployed via zero-shot, few-shot, or fine-tuned transfer.

#### Datasets and benchmarks for wildlife conservation

High-quality datasets are essential for developing and evaluating deep learning models. Wildlife monitoring datasets present unique challenges: extreme class imbalance, variable image quality, taxonomic complexity, annotation expertise requirements, and privacy/legal constraints for endangered species locations. These datasets and their key characteristics are summarized in Table 3.

**Table 3:** Representative datasets for deep learning-based wildlife monitoring

Dataset	Year	Size	Species	Task	Public
Snapshot Serengeti	2015	3.2M images	48	Species ID	Yes
iNaturalist	2021	Millions	∼10,000	Species ID	Yes
Camera Traps	2018	200K images	Multi-sp.	Multi-task	Yes
ATRW (Amur Tiger)	2020	8,076 clips	92 indiv.	Individual Re-ID	Yes
AP-10K (Animal Pose)	2021	10,015 images	54 spp.	Pose Estimation	Yes
Animal Kingdom	2022	50 h video	140+ spp.	Behavior	Yes
APT-36K	2022	36K frames	30 spp.	Pose Estimation	Yes

Note: Datasets are listed in order of publication year. Species counts refer to the number of species or individual identity classes included in the dataset. “Public” indicates datasets available for academic research use without cost (registration or data-use agreement may be required). Dataset sizes reflect figures reported in original publications; continuously updated repositories (e.g., iNaturalist) may have larger current sizes. spp. = species; indiv. = individuals; Multi-sp. = multiple species.

##### Large-scale multi-species benchmarks

Snapshot Serengeti pioneered large-scale camera trap datasets, providing 3.2 million images from Tanzania’s Serengeti National Park labeled with 53 species. This dataset enabled the breakthrough by Norouzzadeh et al.^8^ and remains a primary benchmark. However, geographic restriction limits generalization: models trained on Serengeti often fail elsewhere.^47^ iNaturalist offers massive scale (millions of observations) and global coverage across all taxonomic groups, but crowdsourced labels introduce noise and amateur photography differs substantially from autonomous camera traps.^48^^,^^49^ Camera Trap datasets from multiple locations^50^ address domain shift by providing training data across ecosystems, though smaller scale limits representation of rare species. Trade-offs between scale, diversity, quality, and geographic coverage persist, with no single dataset optimal for all applications. Full metadata for the 35 datasets examined in this review, including dataset name, target taxa, data scale and format, salient features, and access information, are provided in Table S1. The task-type breakdown of these datasets (species recognition, individual re-identification, behavior analysis, and pose estimation) is visualized in Figure S1.

##### Species-specific datasets for endangered wildlife

Targeted datasets for specific endangered species enable fine-grained analysis critical for conservation. The Amur Tiger Re-ID in the Wild (ATRW) dataset^47^ provides 8,076 video clips from 92 individual tigers with stripe pattern annotations, enabling development of tiger-specific re-identification systems achieving >99% accuracy.^18^ Giant panda datasets^18^^,^^50^ include individual IDs, age labels, and behavioral annotations, supporting demographic analysis for population management. Primate face datasets^51^ enable individual recognition and social network analysis for wild chimpanzee communities. PanAf20K^52^ further provides over 200,000 annotated video clips spanning species identity, individual ID, and behavioral categories across multiple wild ape research stations. Marine mammal datasets^53^^,^^54^^,^^55^ provide unique body markings (dorsal fins, spot patterns) for individual tracking across oceanic scales. These specialized datasets drive species-specific model development but limited transferability necessitates separate systems for each target species.

##### Behavioral and pose estimation datasets

Understanding wildlife behavior requires video datasets with temporal action labels and pose annotations. Animal Kingdom^56^ provides over 650 h across 50+ species with hierarchical action labels, enabling multi-species behavior classification. AP-10K^57^ contains 10,015 images from 23 animal families with keypoint annotations for pose estimation across mammals, birds, reptiles, and fish—dramatically expanding beyond prior human/dog-focused datasets. APT-36K^58^ provides 36,000 frames from 30 species with 3D pose annotations. Animal3D^59^ enables 3D reconstruction from monocular video, critical for biomechanical analysis. These datasets reveal substantial architectural differences across taxa (quadrupeds vs. bipeds, terrestrial vs. aquatic), necessitating flexible pose estimation frameworks. However, annotation costs remain prohibitive for comprehensive behavioral coverage across all species of conservation concern. Complete specifications for all species-specific datasets, including geographic coverage and annotation details, are compiled in Table S1.

##### Dataset limitations and future directions

Major datasets^60^^,^^61^^,^^62^^,^^63^^,^^64^ provide benchmarks, while camera trap systems^65^^,^^66^^,^^67^ and biometric databases^47^^,^^50^^,^^68^^,^^69^^,^^70^^,^^71^ enable long-term monitoring and individual re-identification. Despite this progress, current datasets suffer from systematic biases that limit their generalizability, which can be summarized as follows.

**Figure 3:**
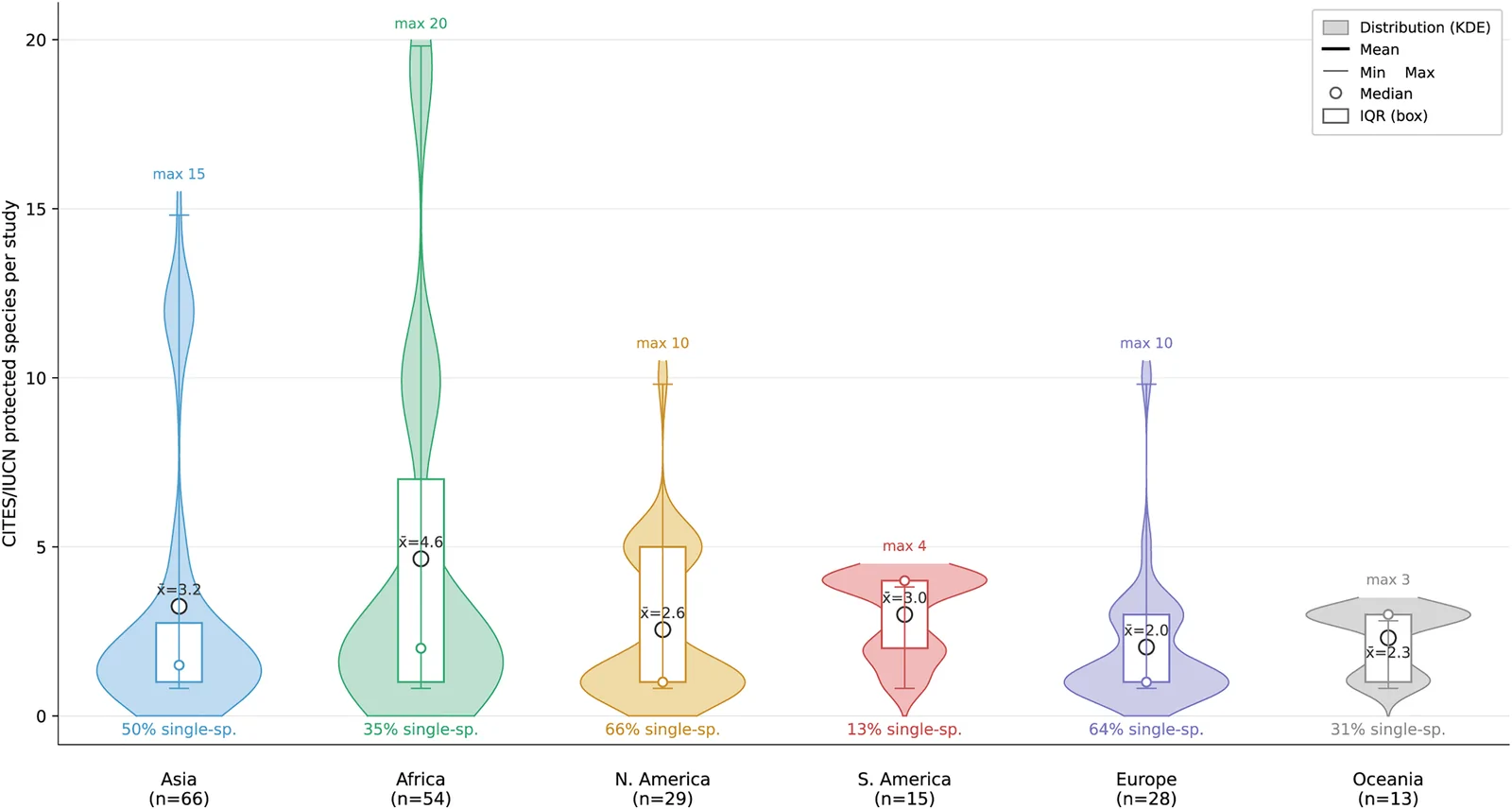
Continental distribution of CITES/IUCN-protected species per wildlife deep-learning study (*n* = 159 studies; general-purpose benchmarks excluded)^56^ Violin plots show the kernel density estimate (KDE) of the number of protected species per study for each continent. Within each violin: the white dot (∘) indicates the median; the colored box spans the interquartile range (IQR, Q1–Q3); whiskers extend to the minimum and maximum observed values. The mean number of protected species per study ($\bar{\text{x}}$) is annotated directly on each violin body. The percentage of single-species studies within each continental corpus is displayed at the base of each violin. General-purpose computer-vision benchmarks were excluded: the Animal Kingdom dataset (850 species, no single geographic attribution) and ImageNet-derived datasets (no clear geographic attribution) are not representative of field-based wildlife research; see STAR Methods for full exclusion criteria. Median values range from 1 to 5 species per study across all six continents; single-species studies constitute 13–66% of each regional corpus.

First, geographic bias is pervasive, as shown in Figure 3. North American and European studies together account for 57 of 159 studies yet show the two narrowest species breadths (median = 2.6 and 2.0 species per study, respectively) and the two highest single-species rates (66% and 64%, respectively). Across all six continents, single-species investigations dominate, constituting 13–66% of each regional corpus. The mean number of protected species per study ranges from 2.0 ± 1.9 (Europe) to 4.6 ± 5.6 (Africa). Africa’s higher mean is mainly driven by a right-skewed distribution (max = 20) from a few multi-species savanna benchmark datasets (e.g., Snapshot Serengeti), not by a general practice of surveying full assemblages. The low single-species proportions in South America (13%) and Africa (35%) similarly reflect a small number of multi-species Neotropical benchmark datasets rather than an established community-level monitoring tradition. These facts indicate that current studies focus on targeted species-specific work. Thus, broad biodiversity surveying remains the exception in wildlife deep-learning research.

Second, taxonomic bias objectively emerges: mammals dominate at 79.8% of reviewed studies (Carnivora 32.7%, Primates 19.5%, Artiodactyla 11.3%, Cetacea & Pinnipedia 8.8%), while Aves account for 10.1%, Reptilia for 5.0%, and Insecta & Invertebrata for only 1.9%. This imbalance is mainly driven by benchmark availability and conservation funding rather than biodiversity or IUCN threat urgency. Table 4 provides a taxonomic performance comparison across DL maturity tiers (2014–2026) to demonstrate this imbalance. As indicated in Table 4, mature groups such as Carnivora and Primates achieve Re-ID accuracies exceeding 90%, supported by dedicated benchmarks (ATRW, ChimpFace) and morphologically distinct individual markings; Cetacea achieves disproportionately strong performance (>85% individual ID) relative to corpus size, owing to decades of pre-DL photo-ID catalog traditions; by contrast, Reptilia, fish, and invertebrates remain at an early or nascent stage—small body size, high species diversity, and prohibitive annotation costs confine the few studies in these groups to species-level classification with no published Re-ID benchmarks. The facts indicate that DL maturity correlates with dataset richness rather than conservation urgency.

**Table 4:** Taxonomic performance comparison across deep learning maturity tiers (2014–2026)

*Taxonomic* *Group*	*Representative Species (IUCN status)*	*n* *Studies*	*Primary DL Task*	*Best Reported* *Accuracy*	*Dominant* *Architecture*	*Key Dataset/Catalog*	*Key Limiting Factors*	*DL* *Maturity*
***▲ Tier I—Mature: Benchmark-rich, High Performance***								

***Carnivora***	***Giant panda (VU), Amur tiger (EN), Snow leopard (VU), Lion (VU), Sun bear (VU)***	52	Re-ID, Species ID, Behavior, Detection	>90% Re-ID (top studies)	CNN + ViT/Swin, YOLO-series	ATRW (tiger), PandaID, ATRW-Snow	Benchmark-rich; annotation cost moderate	★★★★★ Mature
***Primates***	***Chimpanzee (EN), Gorilla (CR), Golden monkey (CR), Drill (EN)***	31	Face Re-ID, Behavior, Pose estimation	85–95% face ID (ChimpFace, Gorilla)	CNN (ResNet/VGG), ViT, DINO	ChimpFace, GorillaWatch, BaboonLand	Occlusion in wild; inter-individual variation	★★★★☆ Advanced

**◆ *Tier II—Intermediate: Disproportionately Strong Given Sample Size***								

***Cetacea &Pinnipedia***	***N. Atlantic right whale (CR), Dolphin* spp. *(VU–EN), Seal (VU)***	14	Individual ID, Fluke/fin matching, Aerial detection	>85% individual ID (right whale catalog)	CNN (ResNet), Siamese nets, ViT	NARWC photo-ID (>700 ind.), NOAA Aerial Survey	Small corpus; occlusion by waves; 3D fluke variation	★★★☆☆ Emerging
***Aves (multiple)***	***Philippine eagle (CR), Wandering albatross (VU), Penguins (EN)***	16	Species classif., Detection (UAV), Counting	75–88% species ID (varies by species)	YOLO-series, CNN (EfficientNet), ViT	iNaturalist birds, KABR (drone), custom aerial	High intra-class variation; camouflage; aerial viewpoint	★★★☆☆ Emerging

***▼ Tier III—Nascent: Severely Underrepresented; No Published Re-ID Benchmarks***								

***Insecta &Invertebrata***	***Monarch butterfly (EN), Apollo butterfly (VU), Rare arthropods (EN)***	3	Species classif. only; no Re-ID	Species-level only (no Re-ID published)	Shallow CNN, MobileNet	iNaturalist insects; no dedicated benchmark	Small body → low resolution; high species diversity; annotation cost prohibitive; no individual markings	★☆☆☆☆ Nascent
***Reptilia***	***Hawksbill sea turtle (CR), Chinese giant salamander (CR)***	8	Species/individual ID (limited)	70–80% species ID (Re-ID nascent)	CNN (ResNet), YOLO (detection)	Sea turtle photo-ID, OBIS records, custom field	Cryptic coloration; underwater occlusion; small labeled corpus	★★☆☆☆ Early
***Actinopterygii***	***Manta ray (EN), Whale shark (EN), Eulachon (threatened)***	5	Individual ID, Species classif.	∼80% spot-pattern ID (manta/whale shark)	CNN (ResNet), Siamese nets	Wildbook (manta), ECOCEAN (w. shark)	Underwater imagery; lighting variation; very small corpus	★★☆☆☆ Early

**Note:** Analysis covers 159 studies published 2014–2026. All species are IUCN-listed as VU, EN, or CR. DL maturity tiers are defined as: ★★★★★ Mature (Re-ID accuracy >90%, dedicated benchmark available); ★★★★☆ Advanced (85–95%, task-specific dataset exists); ★★★☆☆ Emerging (70–85%, growing literature); ★★☆☆☆ Early (<70%, proof-of-concept stage); ★☆☆☆☆ Nascent (species-level classification only, no Re-ID benchmarks published). Performance figures reflect best-reported results across reviewed studies.—= no published benchmark identified. Key findings: DL maturity correlates strongly with benchmark dataset richness and conservation funding, not IUCN threat urgency; Reptilia (100% CR) and Insecta (EN–VU) represent the most critical targets for future dataset investment. Cetacea performance is disproportionately strong relative to corpus size (*n* = 14) owing to decades of pre-DL photo-identification catalog traditions.

##### Third, temporal bias constrains longitudinal inference

Beyond the geographic and taxonomic gaps documented above, long-term datasets tracking population dynamics, range shifts, and behavior adaptations remain scarce despite their conservation value. Privacy concerns further restrict public release of endangered species location data, underscoring the need for FAIR (Findable, Accessible, Interoperable, Reusable) data principles^72^ in conservation science.

##### Future priorities include

(1) Federated learning enabling model training without centralizing sensitive data; (2) synthetic data generation to augment rare species representations; (3) self-supervised learning reducing annotation requirements; and (4) standardized evaluation protocols enabling cross-dataset performance comparison.

### Species recognition and biodiversity assessment

Species recognition—automatically identifying which species appear in images or videos—forms the foundation of automated biodiversity monitoring. Accurate, scalable species recognition enables answering fundamental ecological questions: Which species occupy this area? How do distributions shift seasonally or across years? How do communities respond to environmental changes or conservation interventions?

#### Technical approaches and architectures

##### Classification networks

\When animals are detected or images pre-cropped, species recognition becomes a classification problem: assigning taxonomic labels to visual inputs. Transfer learning from ImageNet^73^^,^^74^ provides powerful initialization even for wildlife domains, despite domain shift between domestic/common species and rare wildlife. Fine-tuning deeper layers while freezing early layers balances leveraging pre-trained features and adapting to wildlife-specific patterns. ResNet variants,^32^^,^^33^ DenseNet,^75^ MobileNet,^76^ and EfficientNet provide accuracy-efficiency tradeoffs that have been widely adopted in published literature. DenseNet^75^ occasionally achieves marginally higher accuracy through feature reuse, valuable for distinguishing similar species. Vision Transformers^7^^,^^39^ and Detection Transformers (DETR)^77^ increasingly appear in recent studies, particularly when sufficient training data exists, offering improved performance on fine-grained recognition tasks.

##### Unified detection-classification pipelines

Real-world camera trap images contain full scenes with animals at various scales, positions, and occlusion levels. End-to-end pipelines combining detection and classification provide automated species recognition from raw camera trap images. YOLO architectures have evolved rapidly from YOLOv1^6^ to YOLO12,^78^ with each version improving speed-accuracy tradeoffs. DETR variants^77^ provide transformer-based alternatives. Recent DETR advances^79^ show competitive real-time performance, while X3D (Cross-3D efficient video network)^80^ enables efficient video recognition, enabling real-time video stream processing. Customized YOLO variants for wildlife^81^^,^^82^^,^^83^ incorporate attention mechanisms emphasizing distinctive body parts, improve small object detection for distant animals, and adapt anchor boxes to typical animal aspect ratios. Similarly, Favorskaya and Pakhirka used a joint CNN with muzzle and body shape features to improve species recognition accuracy.^84^ Two-stage detectors (Faster R-CNN, Cascade R-CNN) provide higher precision (Table 5) when accuracy outweighs speed requirements. Hybrid approaches using fast detection for animal localization followed by high-accuracy classification for species identification optimize accuracy-efficiency tradeoffs. A full comparison of YOLO-series and two-stage detector performance across speed and accuracy metrics is provided in Figure S3.

**Table 5:** Performance comparison of unified detection-classification pipelines for wildlife

Study	Species	Task	Architecture	Performance	Dataset size
Norouzzadeh et al.^8^	48 species	Species ID	ResNet-50	96.6% Acc	3.2M images
Ibraheam et al.^85^	6 species (bear, cougar, deer, elk, moose, mountain goat)	Species Detection	Modified YOLOv2 + DCL	87.2% mAP	∼28,854 training images
Willi et al.^9^	17 species	Species ID	Custom CNN	92.0% Acc	∼450,000 images
Schneider et al.^10^	55 animal classes (+human)	Species ID	DenseNet201	95.6% Top-1 Acc	47,279 images
Shi et al.^86^	Amur Tiger	Individual Re-ID	ResNet-50	93.5% Rank-1	8,076 clips (ATRW)
Wu et al.^14^	Amur Tiger	Individual Re-ID	InceptionResNetV2	99.37% Rank-1	ATRW dataset
Schofield et al.^13^	Chimpanzee	Individual Re-ID	Custom CNN	>90% Rank-1	Video sequences
Nishida et al.^87^	Green turtle (Endangered)	Individual Re-ID	ResNet (fine-tuned)	Improved (>95% baseline)	Collected images
Zang et al.^18^	Giant Panda	Age Group Classification	EfficientNet + Ordinal Regression	85.2–85.99% Acc	∼10,000 images
Renoult et al.^20^	Mandrill (Vulnerable)	Age Estimation (MAE)	DINOv2-L (ViT)	0.58 years MAE	25,500 images (284 individuals)

**Note:** Performance metrics vary by task type, with species identification reported as Top-1 Accuracy (Acc) and individual re-identification as Rank-1 score (R-1) (percentage of queries where the correct match ranks first), making cross-task comparisons indirect. Two-stage detectors such as Faster R-CNN and Cascade R-CNN generally achieve higher precision than single-stage YOLO variants, but at the cost of increased inference time, making them better suited for offline analysis than real-time deployment.

##### Addressing class imbalance

Camera trap datasets exhibit extreme class imbalance, a manifestation of long-tailed recognition challenges prevalent across computer vision^56^^,^^61^^,^^88^: common species (e.g., impala, zebra in African savannas) dominate captures while target endangered species may comprise <1% of images. Standard cross-entropy loss leads models to ignore rare classes. Solutions include: (1) focal loss down-weighting easy examples and emphasizing hard cases, (2) SMOTE (Synthetic Minority Over-sampling Technique)^79^ oversampling minority classes by generating synthetic instances, (3) balanced sampling ensuring equal representation across species during training, and (4) class-weighted loss penalizing rare species misclassification more heavily. For extremely rare species, few-shot learning^89^ (e.g., matching networks,^81^ prototypical networks,^90^ and MAML^91^) as well as data augmentation become essential. Multi-Modal and Temporal Integration. Combining visual data with metadata (capture time, location, temperature, vegetation indices) improves classification, particularly for visually similar species with different temporal/spatial niches. For video data, temporal modeling through 3D CNNs^92^ or Video Transformers^93^ leverages motion cues and behavioral patterns, improving accuracy over single-frame classification while enabling behavior analysis.

#### Real-world applications and performance

##### Savanna ecosystems

Norouzzadeh et al.^8^ achieved 96.6% accuracy across 48 species on the Snapshot Serengeti dataset, matching the performance of crowdsourced human volunteers. Subsequent studies achieved >98% accuracy on dominant species but performance degraded for rare species and challenging conditions (nighttime, occlusion). Extending to unseen locations reveals domain shift: Serengeti-trained models^10^ achieve only 60–70% accuracy on different African ecosystems,^11^^,^^94^^,^^95^ highlighting generalization challenges. Representative species identification accuracy across benchmark studies is summarized in Figure 2A.

##### Forest systems

Dense vegetation, variable lighting, and cryptic species pose distinct challenges. Ibraheam et al.^85^ achieved 87.2% mAP on six forest mammal species using a modified YOLOv2 with DCL, enabling deployment on edge devices for real-time processing. Attention mechanisms highlighting diagnostic features (facial patterns, body markings) proved particularly valuable for distinguishing similar species in low-contrast forest imagery.

##### Asian endangered species

Giant panda recognition^12^ achieved 97.2% accuracy despite similar black-white coloration across individuals, leveraging subtle differences in facial markings and body proportions. Amur tiger detection^96^^,^^97^^,^^98^^,^^99^ using Faster R-CNN achieved 93.5% precision and 92.1% recall, enabling population monitoring without invasive capture for GPS collaring. Snow leopard detection in mountainous terrain^100^^,^^101^^,^^102^ combined CNN recognition with individual identification, enabling mark-recapture population estimates from camera traps alone, with CNN-based detection extended to other CITES-listed Asian species including slow lorises.^103^

##### Marine environments

Underwater imagery presents unique challenges: color distortion, suspended particles, dynamic lighting, variable visibility. Drone-based detection of marine mammals (whales, dolphins, sea turtles) for ship strike avoidance and population surveys achieved 80–90% accuracy^104^^,^^105^ substantially improving upon human observer detection rates while covering vastly larger areas. Beyond aerial surveys, deep learning on very-high-resolution satellite imagery has enabled African elephant detection across heterogeneous landscapes at continental scale, achieving recall exceeding 90%.^106^

#### Cryptic species recognition: Technical limits

Cryptic species—taxa that are morphologically similar but genetically distinct—represent a critical frontier for wildlife AI, exposing two complementary failure modes, both of which are mainly attributed to rarely capturing standard benchmarks.^107^^,^^108^

##### Scottish wildcat vs. domestic cat and hybrids

The Scottish wildcat (Felis silvestris silvestris) is classified as Critically Endangered in the UK, with its survival threatened primarily by introgressive hybridization with feral domestic cats (Felis catus).^109^ The resulting hybrid swarm produces individuals spanning a continuous morphological spectrum between pure wildcat and domestic cat phenotypes, rendering pelage-based field identification unreliable even for experienced researchers.^107^ CNN-based classifiers trained on camera-trap imagery face a compounded difficulty: the diagnostic coat characters—tail banding, dorsal stripe continuity, and flank striping—vary subtly across hybridization grades and are further obscured by variable illumination, occlusion by vegetation, and juvenile developmental stages. Fargetta et al.^107^ demonstrated that even the best-performing CNN architecture achieved only 71% accuracy on a genetically validated test set, compared to 86% on the unvalidated training distribution, revealing a systematic gap between reported benchmark performance and real-world conservation utility. This collapse is partly driven by the continuous nature of hybridization, where diagnostic coat characters shift gradually across hybrid grades rather than separating into discrete classes, a property that standard validation cannot expose.^109^ Critically, misclassification of hybrids as pure wildcats—or vice versa—carries direct legal consequences, as only confirmed wildcats receive statutory protection under UK wildlife law, making false negatives conservation-critical.

These cases share a common lesson: cryptic species discrimination demands uncertainty quantification with expert verification workflows.^110^ That is, models should flag low-confidence predictions for human review rather than defaulting to the majority class, particularly where species identity carries legal consequence for trade enforcement,^111^^,^^112^ statutory protection, or range-overlap surveys.

#### Ecological insights and conservation impact

##### Population trend monitoring

Automated species recognition enables analysis of massive camera trap archives, revealing population trends for multiple species simultaneously. Multi-year studies document range expansions (predator recovery following conservation interventions), seasonal migrations, and responses to climatic variation—insights requiring years of traditional surveys now obtainable from existing camera trap networks.

##### Community composition analysis

Simultaneous monitoring of entire communities reveals predator-prey dynamics, competitive interactions, and biodiversity responses to habitat modification. Automated analysis enables scaling from single sites to landscape-level monitoring, identifying biodiversity hotspots and refugia for targeted conservation.

##### Human-wildlife conflict mitigation

Real-time detection enables proactive conflict prevention. Systems monitoring agricultural boundaries alert farmers to approaching elephants, tigers, or crop-raiding species, enabling non-lethal deterrence.^26^ Roadside cameras detecting animals approaching highways trigger warning signals for motorists, reducing wildlife-vehicle collisions.

##### Efficiency gains and scalability

Automated species recognition reduces identification time from hours per image to milliseconds, enabling analysis of archives containing millions of images. This efficiency gain does not merely accelerate existing analyses—it enables entirely new research questions and management applications requiring population-level data across extensive spatial and temporal scales. Quantified conservation outcomes across 10 taxa—including giant panda monitoring (manual analysis reduced from 12 months to 2 weeks), Amur tiger anti-poaching (92 individuals tracked), African elephant aerial census, and jaguar corridor mapping—are documented in Table S2.

### Individual Re-identification systems

While species recognition answers “what species is present?”, individual re-identification (Re-ID) addresses “which specific individuals?” This capability transforms population ecology by enabling mark-recapture analysis, movement tracking, and social network inference without physical capture, tagging, or invasive procedures.

#### Deep metric learning for animal biometrics

Individual Re-ID exploits the unique natural markings of each species—stripe patterns in tigers and zebras, facial features in primates and bears, spot patterns in leopards and whale sharks, body contours in dolphins and elephants—to establish discriminative biometric identities. The core objective is to learn an embedding space where images of the same individual cluster closely while different individuals are well-separated. Where traditional approaches required manual feature engineering tailored to each species’ marking type, deep metric learning acquires these discriminative features end-to-end from training data. Siamese networks^113^ provided an early and elegant solution, processing image pairs through weight-shared CNNs and outputting similarity scores; they naturally handle both verification (“are these the same individual?”) and identification (“which individual is this?”) tasks, and excel with the limited per-individual training examples common for rare species. Building on this foundation, triplet loss^114^^,^^115^ processes anchor, positive, and negative image triplets simultaneously, pulling same-individual pairs closer while pushing different individuals apart—a formulation that learns more discriminative embeddings than pairwise training.^116^ Hard negative mining, which selects challenging negatives that closely resemble the anchor, further sharpens embedding boundaries in datasets where within-species visual similarity is high.

Two complementary challenges must be addressed once a basic metric space is established: partial occlusion and viewpoint variation. Whole-body embeddings degrade when animals are partially hidden by vegetation, frame edges, or overlapping conspecifics. Part-based approaches address this directly by learning separate embeddings for anatomical regions—head, torso, legs—and combining region-level features for the final matching decision, so that visible parts remain informative even when others are hidden. Independently, camera traps capture the same individual from arbitrary angles, creating apparent appearance variation even between successive sightings of the same animal. Learning pose-invariant representations—through multi-angle training data, augmentation strategies, or explicit 3D reasoning—is therefore equally critical, though multi-view wildlife datasets remain scarce across most species and represent an important direction for future data collection.

#### Species-specific Re-ID solutions

##### Amur tigers: Stripe pattern recognition

These systems enable population monitoring across vast Russian taiga and Chinese forests where physical capture for GPS collaring is logistically impossible. Integration with camera trap networks provides spatially explicit movement data, home range estimates, and population connectivity analysis without invasive procedures. Rank-1 re-identification accuracy across five species is illustrated in Figure 2B. Beyond tigers, deep learning re-identification has been validated across diverse taxa: chimpanzees achieve 92.5% accuracy using video-based facial recognition,^13^ snow leopards reach 98.6% mAP with pose-guided networks,^16^ giant pandas attain 88.6% Rank-1 accuracy with part-based feature fusion,^12^ and hawksbill sea turtles achieve 95.1% using vision transformers on a 13-year longitudinal dataset.^15^^,^^117^

##### Primates: Facial recognition

Primate facial features—eye region patterns, nose shape, facial hair distribution—enable individual recognition analogous to human face recognition but adapted for non-human facial morphology. Schofield et al.^13^ achieved >90% accuracy for chimpanzee facial recognition in videos from Budongo Forest, Uganda, enabling automated behavioral observation and social network inference. The system handles extreme lighting variation, partial occlusion from vegetation, and large pose variation inherent to unconstrained field video. Automated Re-ID enables tracking social interactions, dominance hierarchies, and community dynamics across years without disturbing natural behaviors through close human observation. Similar approaches apply to gorillas,^100^^,^^101^ orangutans, and other great apes with distinct facial features.

##### Marine mammals: Body contour and natural markings

Cetaceans and pinnipeds exhibit unique body markings enabling individual identification from photographs. Dorsal fin shapes and notching patterns provide distinctive features for dolphins^118^ and orcas, while spot patterns enable whale shark identification using algorithms adapted from astronomical pattern matching. Photo-ID databases compiled across decades by research groups worldwide contain tens of thousands of individuals. Automated Re-ID systems mining these archives enable population assessments, migration tracking, and long-term individual life history analysis across ocean basins. Challenges include water surface reflections, variable visibility conditions, and limited photo angles from surface observations.

##### Giant pandas: Facial and body pattern recognition

Despite superficial similarity, giant pandas exhibit subtle individual variation in facial markings and body patterns. Chen et al.^50^ demonstrated individual recognition enabling automated identity inference from camera trap images, supporting population monitoring in fragmented habitats across Sichuan, Shaanxi, and Gansu provinces. Integration with genetic sampling and traditional sign surveys provides comprehensive population assessment, crucial for evaluating reintroduction success and maintaining genetic diversity in isolated subpopulations.

#### Population monitoring applications

##### Mark-recapture without marks

Traditional mark-recapture population estimation requires physical capture to apply uniquely identifiable marks (tags, collars, tattoos), followed by recapture surveys. Automated Re-ID enables “capture” through photographing natural markings, eliminating capture stress, injury risk, and equipment costs while dramatically increasing sample sizes. Statistical frameworks (spatially explicit capture-recapture, robust design) originally developed for physical marks apply directly to photo-identification data, enabling abundance estimation, survival analysis, and recruitment inference.

##### Movement and space use

Multi-site camera trap networks with individual identification provide spatially explicit movement data. Re-ID across camera locations reveals home ranges, dispersal patterns, habitat connectivity, and population fragmentation. For example, tiger Re-ID across reserve boundaries documents transboundary movements requiring coordinated international conservation, while panda Re-ID across isolated habitat patches quantifies population connectivity essential for genetic management.

##### Social structure and behavior

Repeated observations of identified individuals enable inferring association patterns, dominance hierarchies, and social networks. Primate Re-ID facilitates automated behavioral observation previously requiring years of researcher presence habituating animals to human observers.^56^ Social network analysis reveals keystone individuals, information flow patterns, and social learning pathways critical for conservation translocation planning and predicting population responses to individual removals.

##### Long-term individual life histories

Multi-year Re-ID provides longitudinal data on individual survival, reproduction, and life history trajectories. Age-structured population models parameterized with individual-based data provide more reliable population projections than cross-sectional surveys. Long-term marine mammal photo-ID catalogs spanning decades document reproductive success, senescence, and responses to environmental change at individual resolution.

### Demographic analysis via soft biometrics

Individual identity answers “who?” but population management also requires “how old?” and “male or female?” This demographic information—age and sex structure—reveals population health, reproductive potential, and conservation priorities. Soft biometrics extracts these attributes from visual data without physical examination.

#### Age and sex estimation methods

Age estimation from visual data must respect the inherent structure of the problem: unlike arbitrary categorical labels, ages carry a natural ordering, and errors should reflect this ordering (predicting 5 years when the true age is 3 is far less serious than predicting 50). Figure 2C compares mean absolute error (MAE, in years) across four wildlife studies, with values also expressed relative to mean species lifespan to enable cross-taxon comparisons. Freytag et al.^17^ applied Log-Euclidean CNNs to chimpanzee facial images (MAE = 3.6 years; 8.0% of lifespan), while Zang et al.^18^ exploited ordinal regression on giant panda facial images (MAE = 2.4 years; 12.0%). Renoult et al.^20^ fine-tuned DINOv2-Large on 25,500 mandrill portraits, achieving MAE = 3.3 years (16.5%), the highest relative error across the four studies. The most precise result came from Zhao et al.,^19^ whose dual-branch model combining facial appearance with nose melanin patterns yielded MAE = 0.568 years for Amur tigers (4.1%)—demonstrating that species-specific biometric features can dramatically improve precision. Notably, relative error is the more informative cross-species metric: the mandrill’s MAE of 3.3 years (16.5%) represents substantially poorer performance than the chimpanzee’s larger absolute MAE of 3.6 years (8.0%). Where continuous age estimation remains unreliable due to camera-trap image quality constraints, age category classification (juvenile, subadult, adult, senior) provides ecologically actionable demographic information with greater practical robustness.

Sex classification draws on the visible morphological differences between sexes: size dimorphism, coloration, and anatomical features such as antlers or manes provide visual cues that multi-task CNNs can learn jointly alongside species and age attributes, improving efficiency through shared feature representations.^119^^,^^120^ The achievable accuracy, however, varies widely with the degree of sexual dimorphism; highly dimorphic species such as gorillas permit reliable automated sexing, while morphologically similar sexes may remain indistinguishable from imagery alone. In such cases, correlated proxy variables extend the reach of demographic inference: body condition scores reflecting nutritional status, behavioral context (adults accompanied by offspring are likely female in species with maternal care), and ensemble integration of multiple visual and contextual cues can together yield population-level demographic estimates even when direct sex or age determination from a single image is not possible.

#### Applications in population structure analysis

##### Giant panda demographic monitoring

Zang et al.^18^ predicted giant panda ages from facial images using machine learning, achieving 85–90% accuracy for age category classification (juvenile, subadult, adult). This enables inferring age structure from camera trap surveys, revealing recruitment rates and population aging, while also reducing manual analysis time from 12 months to 2 weeks and enabling real-time population tracking (Table S2); architecture-level accuracy comparisons are illustrated in Figure S2. Li et al.^121^ analyzed pregnancy status through behavioral hierarchies and body appearance, providing real-time reproductive success monitoring without invasive examination or den monitoring.

##### Sex ratio assessment

Population sex ratios influence reproductive potential and extinction risk. Male-biased adult sex ratios, common in heavily hunted populations, reduce reproductive rates. Automated sex determination from camera traps enables efficient sex ratio assessment without capture-based sexing requiring invasive examination or molecular techniques. For sexually dimorphic megafauna (elephants, ungulates), automated methods achieve >90% accuracy, enabling population-scale sex ratio inference.

##### Recruitment and survival estimation

Age structure reveals population dynamics: high juvenile ratios indicate successful recruitment, while aging populations suggest reproductive failure or juvenile mortality. Long-term camera trap datasets with age classification enable cohort analysis, tracking birth year classes through time to estimate age-specific survival—critical parameters for population viability analysis. Deviations from stable age distributions signal emerging conservation problems requiring intervention.

##### Reproductive success monitoring

Presence of juveniles with adults indicates recent successful reproduction. Temporal analysis of adult-juvenile associations estimates breeding season timing, litter sizes, and offspring survival—particularly valuable for elusive species with cryptic reproductive behavior. Integration with individual ID enables per-capita reproductive success estimation and identifying productive versus non-reproductive individuals.

#### Integration with conservation management

##### Adaptive management feedback

Real-time demographic monitoring enables adaptive management: rapidly detecting population declines, recruitment failures, or sex ratio skews and implementing corrective actions. For example, detecting high juvenile mortality triggers investigation of causal factors (predation, disease, nutritional stress), guiding targeted interventions. Monitoring post-translocation demographics assesses reintroduction success and adaptive management of release strategies.

##### Prioritization of conservation resources

Demographic data identifies conservation priorities: populations with strong recruitment and balanced sex ratios require monitoring but may be lower priority for intensive intervention than aging, recruitment-failing populations requiring active management. Age-sex structured population models parameterized with monitoring data project population trajectories under alternative management scenarios, optimizing resource allocation.

##### Translocation and reintroduction planning

Successful translocations require demographically balanced founder populations. Automated demographic assessment of source populations guides individual selection, ensuring appropriate age-sex composition for establishing viable populations. Post-release monitoring documents demographic development, revealing bottlenecks and informing adaptive management.

##### Climate change response tracking

Demographic responses (recruitment failure, sex ratio skews due to temperature-dependent sex determination) provide early warning of climate change impacts. Long-term demographic monitoring across environmental gradients identifies vulnerable populations and informs climate adaptation strategies.

### Behavioral understanding and pose estimation

Understanding what animals do—their behaviors, activities, and interactions—provides insights into welfare, ecology, and conservation needs beyond distribution and demographics. Automated behavioral analysis from video enables continuous observation at scales impossible through human observation.

#### Action recognition architectures

Recognizing behavior from video is fundamentally a spatiotemporal problem: models must learn not only what is present in a scene (appearance) but also how it changes over time (motion). Feng et al.^119^ utilized Two-Stream networks, processing RGB frames for appearance through one pathway and optical flow for motion through a second before fusing both—establishing that explicit motion modeling reliably outperforms single-frame classification. A natural progression was to integrate the two streams from the outset: 3D CNNs^92^ apply convolutions across both spatial and temporal dimensions simultaneously, learning hierarchical spatiotemporal representations end-to-end. C3D (Convolutional 3D), I3D (Inflated 3D), and their successors demonstrated substantial gains over Two-Stream approaches, effectively capturing stereotyped behaviors such as grooming, feeding, and locomotion through learned motion patterns.

The computational cost of 3D convolutions—roughly 100× that of 2D equivalents—motivated more efficient designs. SlowFast networks^122^^,^^123^^,^^124^^,^^125^^,^^126^ address this by splitting processing across two temporal pathways: a slow pathway at low frame rate captures spatial semantics, while a fast pathway processes high frame rates for fine-grained motion dynamics, achieving strong performance at reduced compute—a balance important for resource-constrained field deployments. For behaviors that unfold over longer timescales, however, local convolution windows are insufficient; Video Transformers^93^ address this through global self-attention across frames, excelling at complex multi-step behaviors such as social interactions and extended foraging sequences, as demonstrated by PandaFormer for wild giant panda behavior recognition.^127^ Representative mean average precision (mAP) values across recent wildlife behavior recognition studies are summarized in Figure 2D. A further dimension of information becomes available when visual data can be paired with complementary modalities: combining video with acoustic signals,^128^ accelerometry from GPS collars, or environmental metadata substantially improves behavior classification and can reveal behavioral states invisible in imagery alone, though the multi-sensor datasets required for fusion training remain scarce in wildlife contexts. Multimodal fusion combining visible-spectrum and thermal infrared imagery (RGB-T) offers a promising complement to acoustic and accelerometric modalities, particularly for nocturnal species detection.^24^^,^^129^ Benchmark trends across these architectures are illustrated in Figure S2, with^130^ providing a detailed comparison of X3D, SlowFast, and transformer-based models on the *in situ* KABR dataset of wild ungulate behavior.

#### Pose estimation for wildlife

##### Anatomical keypoint detection

Pose estimation localizes anatomical keypoints (joints, facial features, body landmarks), providing skeletal representations encoding posture and body configuration. Early wildlife pose estimation adapted human pose methods (OpenPose^131^ HRNet^132^), but animal morphological diversity requires species-specific adaptations. Subsequent works—such as DANNCE,^133^ DeepLabCut,^134^ LEAP,^135^ animal-adapted OpenPose^136^ and HRNet-based variants^137^—enable multi-species pose estimation. Large-scale datasets such as AP-10K^57^ and APT-36K^58^ further support this task. Recent advances include Animal3D,^59^ AniMer,^138^ ScarceNet,^139^ and transformer-based approaches,^139^ enable multi-species pose estimation.

##### Animal-specific datasets and models

AP-10K^57^ provides 10,015 images with keypoint annotations for 23 animal families and 54 species, dramatically expanding pose estimation beyond humans, cats, and dogs. APT-36K^58^ contains 36,000 annotated frames from 30 species. These datasets reveal substantial architectural variation across taxa (number of limbs, body plans), motivating flexible architectures accommodating diverse animal body structures. Part affinity fields, originally developed for multi-person pose estimation, adapt naturally to multi-animal scenarios in group-living species.

##### 3D pose and shape estimation

Monocular 2D pose provides limited information for biomechanical analysis requiring 3D joint positions and orientations. Animal3D^61^ enables 3D reconstruction from single-view video through learned shape priors and temporal consistency constraints. 3D pose estimation enables quantitative gait analysis, biomechanical modeling of locomotion, and precise behavioral quantification (e.g., distance traveled, movement speeds) from camera trap video—previously requiring specialized motion capture or GPS tracking.

##### Challenges and limitations

Wildlife poses estimation faces significant challenges: articulated bodies with many degrees of freedom, self-occlusion, fur/feathers obscuring anatomical landmarks, variable image quality, and diverse viewing angles. Most published work focuses on captive animals with controlled lighting and close viewing distances; field deployment in natural habitats with unconstrained camera trap imagery remains challenging. Transfer learning from human or domestic animal models provides limited benefit for phylogenetically distant species with fundamentally different morphology.

#### Behavioral ecology applications

##### Activity budget analysis

Quantifying time allocation across behaviors (foraging, resting, traveling, social interaction) provides insights into energetics, habitat quality, and disturbance impacts. Automated activity classification from continuous video enables 24-h activity monitoring previously requiring prohibitively expensive human observation. Diurnal activity patterns, seasonal shifts in time budgets, and behavioral responses to human presence become quantifiable at population scale.

##### Stereotyped behavior detection in captivity

Captive wildlife often develop abnormal repetitive behaviors (stereotypies) indicating suboptimal welfare. Automated stereotypy detection^140^ enables continuous welfare monitoring, identifying individuals requiring enrichment intervention and assessing environmental modification effectiveness. This application has seen strong uptake in zoos and sanctuaries for improving animal welfare through data-driven management.

##### Social behavior and interaction networks

Pose estimation enables detailed interaction analysis: identifying physical contact, aggressive displays, affiliative behaviors, and spatial relationships. Temporal pose dynamics reveal nuanced social behaviors: approach trajectories, turn-taking in vocal exchanges, coordinated group movements. Bain et al.^128^ demonstrated automated recognition of wild chimpanzee social behaviors from audiovisual data, enabling long-term social network analysis without human observers habituating the group—particularly valuable for endangered species where observer presence risks disturbance.

##### Movement ecology and habitat use

Fine-scale movement patterns from pose tracking provide insights into habitat use beyond camera trap “presence/absence” data. Gait characteristics, movement speeds, and turning behaviors reveal energetic costs of movement across habitat types, indirect effects of predation risk on vigilance behaviors, and responses to anthropogenic disturbance. 4D pose enables quantitative biomechanical analysis of locomotion costs across heterogeneous landscapes.

##### Locomotion analysis for biomechanics and welfare

Detailed pose dynamics enable gait analysis, lameness detection, and assessment of locomotory health. For rehabilitated wildlife pre-release, locomotion analysis confirms functional recovery before release to the wild. For captive breeding programs, locomotion assessment identifies musculoskeletal problems requiring intervention. For wild populations, gait analysis can detect injury or disease at population scale, providing early warning of health problems.

##### Integration with species recognition and Re-ID

Combining species recognition, individual Re-ID, and behavior classification provides comprehensive understanding: who is doing what, when, and where. Longitudinal behavior profiles for identified individuals enable linking behavioral variation to reproductive success, survival, and social status—connecting individual-level processes to population dynamics and informing behavioral ecology theory with unprecedented sample sizes and temporal coverage.

## Discussion

### Challenges and optimization strategies

Despite remarkable progress, deploying robust deep learning systems for wildlife monitoring faces persistent challenges that are not independent: they interact and compound in ways that make real-world deployment substantially harder than benchmark performance suggests. Data scarcity for rare species and extreme class imbalance is often inseparable—the most threatened taxa are precisely those with fewest training examples. Domain shift then emerges as a second-order obstacle: even when a model is trained on sufficient data, it may fail at a new deployment site because the training distribution does not match field conditions. The following analysis examines these challenges in sequence, maps them to proven optimization strategies (summarized in Table 6), and highlights where interactions between challenges require multi-pronged solutions.

**Table 6:** Key challenges in wildlife monitoring and corresponding deep learning optimization strategies

Challenge	Optimization strategy	Representative methods	Typical improvement
Data Scarcity	Transfer Learning, Few-shot Learning, Data Augmentation	Meta-learning, Self-supervised pre-training	+10–20%
Class Imbalance	Focal Loss, Balanced Sampling, Class Weighting	RetinaNet Focal Loss, SMOTE	+15–25%
Domain Shift	Domain Adaptation, Multi-site Training	Adversarial Training, Test-time Adaptation	+20–30%
Environmental Interference	Data Augmentation, Robust Architectures	MixUp, CutOut, AutoAugment	+5–15%
Computational Constraints	Model Compression, Knowledge Distillation, Pruning	MobileNet, EfficientNet, Quantization	5–10× speedup
Individual Variation	Part-based Models, Multi-view Training	Pose Normalization, Temporal Aggregation	+10–15%

**Note:** Performance improvements are approximate ranges reported across multiple studies; actual gains depend on baseline methods and specific datasets. “×” denotes speedup relative to the uncompressed baseline.

#### Environmental interference and robust feature learning

Camera trap imagery exhibits extreme environmental variability: lighting ranges from harsh midday sun through twilight to complete darkness with infrared illumination; weather introduces rain, fog, snow, and dust affecting visibility; vegetation creates occlusion, background clutter, and camouflage. These factors substantially degrade model performance trained on constrained datasets. Solutions include: (1) data augmentation simulating environmental variability through photometric transformations (brightness, contrast, color jittering), geometric transformations (rotation, scaling, cropping), and synthetic occlusions^141^ (2) domain randomization during training to prevent overfitting to specific environmental conditions, (3) multi-domain training incorporating data from diverse locations and conditions^50^ and (4) robust architectures with attention mechanisms focusing on discriminative features invariant to environmental nuisance factors.

#### Data scarcity for rare species

The most threatened species often have minimal training data: few camera trap captures, limited geographic sampling, and insufficient examples of rare behaviors. Standard supervised learning requires thousands of labeled examples per class—infeasible for many endangered species. Solutions include: (1) transfer learning from abundant related species, leveraging evolutionary similarity in visual features (2) few-shot learning meta-learning approaches^92^^,^^142^^,^^143^ enabling model adaptation from <10 examples per species, (3) data augmentation and synthetic data generation through generative models, including GANs^144^ and diffusion-based approaches, to augment rare species representations; consistency models and denoising generative models offer additional augmentation pathways for rare species. (4) self-supervised pre-training on unlabeled camera trap images learning generic wildlife features^118^ and (5) crowdsourcing labels through platforms such as Zooniverse, though label quality control becomes critical.

#### Class imbalance and long-tailed distributions

Real-world species distributions follow power laws: few abundant “background” species dominate captures while target endangered species comprise <1% of images. Standard training prioritizes abundant classes, causing models to essentially ignore rare species. Solutions include: (1) focal loss mathematically down-weighting easy examples and emphasizing challenging cases, (2) class-balanced sampling ensuring equal training representation across species regardless of capture frequency, (3) class-weighted loss functions assigning higher penalty to rare species misclassification, (4) two-stage training: first learning general features from abundant data, then fine-tuning on rare species with balanced sampling, and (5) ensemble methods combining generalist models trained on all data with specialist models trained specifically on rare species.

#### Domain shift and cross-location generalization

Models trained at one location often dramatically underperform at new locations despite recognizing the same species.^47^ Domain shift arises from differences in: camera models/settings, habitat structure, lighting conditions,^145^ animal subspecies appearance, and background context. Serengeti-trained models achieve only 60–70% accuracy on different African reserves, compared to >96% within Serengeti. Solutions include: (1) domain adaptation techniques aligning feature distributions across source (training) and target (deployment) domains;^50^ (2) multi-site training incorporating data from diverse locations, accepting lower single-site performance for better generalization; (3) domain-invariant feature learning through adversarial training; and (4) test-time adaptation updating models using unlabeled target domain data without retraining from scratch.

#### Individual variation and pose handling

Animals exhibit substantial within-individual variation: pose, viewing angle, age progression, seasonal appearance changes (pelage, antler growth), body condition variation. Re-ID systems must maintain intra-individual similarity while preserving inter-individual discrimination. Solutions include: (1) part-based models robust to partial visibility and pose variation (2) multi-view training data showing individuals from diverse angles, (3) pose normalization through learned spatial transformers or explicit 4D reasoning, (4) temporal aggregation: combining predictions across multiple sightings improves reliability beyond single-image classification, and (5) longitudinal training data tracking individuals across years enables learning appearance variation over time.

#### Computational constraints and edge deployment

Conservation applications often require real-time processing in resource-constrained environments: solar-powered edge devices, drones with limited battery life, field deployments without cloud connectivity. Standard deep learning models are computationally expensive and power-hungry. Solutions include: (1) efficient architectures—MobileNets, EfficientNets GhostNet, ShuffleNet, SqueezeNet and model compression techniques, including quantization, binary networks,^146^ knowledge distillation,^147^ pruning^148^ and hardware acceleration^149^—designed for mobile deployment, (2) model compression through pruning quantization to low-precision weights, and knowledge distillation training small “student” models to mimic large “teacher” models, (3) neural architecture search automatically discovering optimal accuracy-efficiency tradeoffs, (4) cascade approaches using fast models for initial filtering then applying accurate-but-expensive models only to detected animals, and (5) edge-cloud hybrid architectures performing lightweight processing locally with selective cloud uploads for complex cases.

#### Environmental and ethical considerations

The application of AI to environmental challenges requires careful consideration of both opportunities and risks. Energy consumption of large model training, carbon footprints of continuous monitoring systems, and equitable access to conservation technology demand attention. Community-based monitoring approaches offer pathways to ensure local stakeholder engagement and data sovereignty. Deploying lightweight CNN models on IoT-enabled edge devices^150^^,^^151^^,^^152^ enables resource-efficient field applications while reducing environmental impact.

### Future directions and emerging technologies

#### Foundation models for wildlife monitoring

Large-scale pre-training on massive diverse datasets has produced foundation models with remarkable zero-shot and few-shot capabilities. CLIP learns joint vision-language representations, enabling open-vocabulary recognition: describing novel species in text and immediately recognizing them visually without any species-specific training. SAM achieves zero-shot segmentation from minimal prompts, enabling precise animal segmentation without training data. For wildlife conservation, foundation models offer: (1) rapid adaptation to new species with minimal labeled data through prompt tuning or LoRA fine-tuning, (2) unified frameworks handling diverse species without separate species-specific models, (3) open-vocabulary systems accommodating taxonomic updates and cryptic species complexes without architectural changes, and (4) multi-task capabilities (detection, segmentation, classification) from single models. However, current foundation models exhibit domain shift on wildlife imagery: generic models pre-trained on web images struggle with camera trap aesthetics, nighttime infrared, unusual viewing angles, and camouflaged animals. Research priorities include wildlife-specific pre-training, efficient domain adaptation methods, and understanding foundation model failure modes on conservation-critical edge cases.

#### Multimodal learning and sensor fusion

Real-world monitoring increasingly combines visual data with complementary modalities: acoustic monitoring (bioacoustics detecting vocalizations), environmental sensors (temperature, vegetation indices from multispectral imagery), movement data (GPS, accelerometry from collars), and spatial context (habitat maps, anthropogenic pressure layers). Multimodal fusion promises richer understanding than vision alone: identifying visually cryptic species through vocalizations,^134^ improving classification by incorporating ecological context^11^ and enabling behavior inference from multi-sensor data.^153^ Challenges include developing effective fusion architectures, handling missing modalities during deployment, and creating multimodal training datasets pairing diverse sensor streams with ground truth labels. Contrastive learning frameworks such as CLIP demonstrate powerful approaches for learning joint representations across modalities, offering templates for wildlife-specific multimodal models.

#### Spatiotemporal modeling for behavioral ecology

Most current approaches process images or short video clips independently, ignoring rich temporal context. Spatiotemporal models^41^^,^^104^^,^^105^^,^^154^^,^^155^^,^^156^ explicitly representing animal trajectories, long-term behavioral sequences, and interaction dynamics enable understanding beyond static recognition: inferring social relationships^157^ from repeated co-occurrences, detecting territorial behaviors from movement patterns, and identifying disturbance responses from behavioral shifts. Graph neural networks representing animals as nodes and interactions as edges provide natural frameworks for social network inference from observational data. Challenges include computational scaling to long sequences, handling variable observation densities across time, and developing ecologically meaningful evaluation metrics beyond computer vision benchmarks.

#### Self-supervised learning for unlabeled data

Conservation databases contain vast unlabeled camera trap archives: millions of images from long-term monitoring, but lacking species labels due to annotation costs. Self-supervised learning^118^ extracts useful representations from unlabeled data through pretext tasks requiring no labels (e.g., predicting image rotations, reconstructing masked image regions, contrastive learning pulling augmentations of the same image together). Self-supervised pre-training on unlabeled camera trap images followed by supervised fine-tuning on labeled data substantially improves performance, particularly for rare species with limited labels. Future research should explore wildlife-specific self-supervised objectives, active learning strategies identifying most informative images for labeling, and semi-supervised approaches leveraging both labeled and unlabeled data simultaneously.^158^

#### Efficient edge AI and sustainable computing

The environmental cost of deep learning—energy consumption for training and inference—contradicts sustainability principles central to conservation. Edge deployment reduces this cost by enabling local processing without cloud communication, but requires ultra-efficient models. Recent advances include aggressive quantization (2-bit, ternary weights), dynamic inference adjusting computation based on input complexity, and neural architecture search discovering optimal efficiency frontiers. Brain-inspired computing (neuromorphic chips, spiking neural networks) promises orders-of-magnitude efficiency improvements, particularly for continuous video processing. Sustainable AI practices including carbon-aware training (scheduling training during renewable energy availability) and model sharing (reusing pre-trained models rather than training from scratch) should become standard in conservation technology development.

### Limitations

This review has several limitations. First, our focus on peer-reviewed literature (2014–2026) may underrepresent rapidly evolving techniques from recent preprints and domain-specific knowledge from conservation practitioners. Second, English-language publication bias may exclude important work in other languages, particularly from biodiversity-rich regions. Third, our categorization into four technical domains (species recognition, individual Re-ID, soft biometrics, behavior analysis) imposes artificial boundaries on increasingly integrated systems. Fourth, our emphasis on threatened species may not represent broader wildlife monitoring applications. Fifth, rapid field evolution means cutting-edge methods may not yet have peer-reviewed publications documenting deployment outcomes. Finally, our focus on algorithmic and technical advances may underemphasize critical socio-technical challenges: data sharing policies, computational resource access in low-resource settings, integration with traditional ecological knowledge, and equitable benefit-sharing from technologies developed using data from biodiversity-rich regions.

### Conclusions

Deep learning has fundamentally transformed wildlife monitoring, converting once-intractable volumes of camera trap imagery into actionable conservation intelligence across taxa, ecosystems, and continents. From species recognition achieving expert-level accuracy across diverse taxa and challenging conditions, through individual re-identification enabling mark-recapture without physical marks, to demographic inference and comprehensive behavioral understanding—these technologies provide unprecedented insights for conservation decision-making.

Key achievements include: (1) Robust species recognition: systems achieving >95% accuracy across 48+ species,^8^ enabling automated processing of millions of camera trap images and dramatically reducing monitoring costs. (2) Individual tracking without tagging: Re-ID systems achieving >99% accuracy for tigers^18^ >90% for primates,^56^ enabling population assessment and movement ecology without invasive capture and marking. (3) Automated demographic analysis: age/sex estimation from visual data providing population structure information traditionally requiring physical examination.^43^ (4) Comprehensive behavioral understanding: action recognition and pose estimation enabling 24/7 activity monitoring, social network inference, and welfare assessment at population scale.^66^^,^^67^^,^^134^^,^^159^

Critical challenges persist. Data scarcity for rare species requires developing few-shot learning approaches and leveraging foundation models’ transfer capabilities. Domain shift across locations demands improved generalization through multi-site training and domain adaptation. Computational constraints for field deployment necessitate continued model compression and efficient architecture development. Integration into conservation workflows requires bridging computer vision research and conservation practice through interdisciplinary collaboration.

Emerging opportunities promise transformative advances. Foundation models (CLIP, SAM, DINO) offer open-vocabulary recognition and zero-shot capabilities, enabling rapid adaptation to novel species without species-specific training. Multimodal learning combining vision with acoustics, GPS, and environmental data provides richer ecological understanding than vision alone. Advanced spatiotemporal modeling captures behavioral dynamics, social interactions, and movement patterns essential for understanding population processes. Self-supervised learning extracts useful representations from vast unlabeled camera trap archives, addressing annotation bottlenecks. Efficient edge AI enables real-time processing in resource-constrained field settings while reducing environmental costs of computation.

Success requires sustained collaboration between computer vision researchers and conservation practitioners, combining technical innovation with ecological insight. Dataset sharing, reproducible research practices, and open-source tool development accelerate progress while ensuring equitable access. Algorithmic development should be guided by conservation priorities: robustness over marginal accuracy gains, computational efficiency for deployment accessibility, interpretability for building practitioner trust and regulatory acceptance, and integration with existing monitoring programs.

As biodiversity loss accelerates, automated monitoring becomes increasingly urgent. The technologies reviewed here provide essential tools for understanding, protecting, and restoring wildlife populations. Future progress depends on continued algorithmic innovation, expanded datasets covering underrepresented species and regions, practical deployment in resource-limited settings, and genuine partnerships between technological and conservation communities. The path forward is clear: from pixels to conservation impact, from algorithmic advances to species saved—deep learning offers powerful capabilities, but realizing conservation impact requires intentional efforts to translate research into accessible, deployable tools addressing real-world conservation challenges.

## Resource availability

### Lead contact

Requests for further information and resources should be directed to and will be fulfilled by the lead contact, Nian Cai (cainian@gdut.edu.cn).

### Materials availability

This study did not generate new unique reagents.

### Data and code availability

This review analyzes published literature. All cited studies include information on data and code availability. Major datasets are publicly available through the repositories listed in the key resources table. Custom analysis code will be made available upon reasonable request to the lead contact.

## Acknowledgments

This work was supported by the 10.13039/501100001809National Natural Science Foundation of China (grant no. 62171142), the 10.13039/501100019394CUI CAN Program of Guangdong Province, China (grant no. CC/XM-202402ZJ0601), and the Guangzhou Nanfang University School-level Project (grant no. 2025XK031).

## Author contributions

D.X.: conceptualization, methodology, investigation, formal analysis, data curation, visualization, and writing – original draft. Y.C.: investigation, validation, data curation, and writing – review and editing. M.X.: methodology, software, validation, formal analysis, and writing – review and editing. C.L.: resources, supervision, and writing – review and editing. N.C.: funding acquisition, project administration, supervision, validation, and writing – review and editing.

## Declaration of interests

The authors declare that they have no known competing financial interests or personal relationships that could have appeared to influence the work reported in this paper.

## STAR★Methods

### Key resources table

**Table:** 

REAGENT or RESOURCE	SOURCE	IDENTIFIER
**Datasets**		

Snapshot Serengeti	Swanson et al.^60^	https://lila.science/datasets/snapshot-serengeti
LoTE-Animal	Liu et al.^137^	https://lote-animal.github.io/
Camera Traps Dataset	Beery et al.^94^	https://lila.science/datasets
ATRW (Amur Tiger)	Li et al.^47^	https://cvwc2019.github.io
AP-10K (Animal Pose)	Yu et al.^57^	https://github.com/AlexTheBad/AP-10K
Animal Kingdom	Ng et al.^56^	https://sutdcv.github.io/Animal-Kingdom

**Software**		

PyTorch	Facebook AI Research	https://pytorch.org
TensorFlow	Google Brain	https://tensorflow.org
DeepLabCut	Mathis et al.^134^	https://www.mackenziemathislab.org/deeplabcut
Wild Me Platform	Parham et al.^65^	https://www.wildme.org/
MegaDetector	Beery et al.^94^	https://github.com/microsoft/Biodiversity

### Method details

The systematic review methodology underpinning the findings reported in the Results section—including database search strategy, inclusion and exclusion criteria, and data extraction procedures—is described in full below. The 159 primary research studies (from a total of 197 retrieved papers) synthesized in this review were identified and selected according to the protocol detailed in the Literature Analysis Framework, Literature Search and Selection, and Data Extraction and Coding subsections that follow. The remaining 38 retrieved papers consisted of reviews, methodological frameworks, or dataset descriptions that did not meet the inclusion criteria for quantitative synthesis, though they were retained for contextual discussion where applicable.

#### Systematic literature search and selection criteria

We systematically searched Web of Science Core Collection, Google Scholar, IEEE Xplore, and arXiv for literature published between January 2014 and January 2026. Boolean search strings combined deep learning methodologies (e.g., “convolutional neural networks”, “object detection”, “Vision Transformer”, “attention mechanism”, “foundation models”, “state space models”) with wildlife monitoring applications (e.g., “species recognition”, “individual identification”, “camera trap”, “endangered species”, “pose estimation”, “behavior recognition”). Database searches were supplemented by backward and forward citation tracking of key papers and by expert consultation to identify additional relevant work.

Inclusion criteria were: (1) peer-reviewed journal articles (SCI/SSCI indexed) or proceedings from major computer vision and machine learning conferences (e.g., CVPR, ICCV, ECCV, NeurIPS, ICLR, AAAI) as well as highly cited preprints (>50 citations); (2) studies involving CITES-listed species or IUCN Red List categories (Critically Endangered, Endangered, Vulnerable, Near Threatened) or nationally protected species; (3) empirical validation with quantitative performance metrics on real-world wildlife data (not purely synthetic); (4) sufficient methodological detail to allow reproducibility.

Exclusion criteria removed studies focusing exclusively on domestic animals, livestock, or common pest species without conservation relevance; traditional computer vision approaches not employing deep learning; purely theoretical work without empirical validation; duplicate publications (conference and journal versions counted once, favoring the journal version); and papers with clear methodological flaws (e.g., inappropriate train/test splits, unreported metrics, inflated performance claims).

After title/abstract screening of over 800 candidate papers, we conducted full-text review of 350 papers. A total of 159 primary studies met all inclusion criteria and form the empirical basis of this review. An additional 38 references—covering foundational deep learning architectures (e.g., CNNs, ViT), recent representative models (e.g., Mamba, CLIP, SAM), benchmark datasets, and prior review papers—were cited to provide technical context and are not subject to the systematic inclusion criteria above.

#### Data extraction and coding

For each of the 159 included studies, we systematically extracted the following categories:

**Dataset Characteristics** – dataset name, source, availability (public/restricted); number of images/videos and temporal span; species coverage and taxonomic diversity; geographic scope and habitat types; annotation types (species labels, individual IDs, bounding boxes, keypoints, behavior labels); data collection methodology (camera traps, drones, handheld cameras, video surveillance); class distribution and imbalance statistics.

**Methodological Details** – network architecture (base model, customizations, parameter count); training procedures (optimizer, learning rate schedule, batch size, data augmentation); pre-training strategy (ImageNet initialization, domain-specific pre-training, training from scratch); loss functions; computational requirements (GPU hours, memory).

**Performance Metrics** – classification: accuracy, top-k accuracy, precision, recall, F1-score, per-class performance; detection: mAP, precision-recall curves, IoU thresholds, inference speed (FPS); re-identification: Rank-1 accuracy, mAP, CMC curves; regression (e.g., age estimation): MAE, RMSE, age category accuracy; video understanding: frame-level accuracy, video-level accuracy, temporal IoU.

**Application Context** – target species and conservation status; ecological questions addressed; geographic deployment location; real-world validation (lab-only vs. field-deployed); conservation impact (population estimates, management decisions informed); integration with existing monitoring programs.

**Challenges and Limitations** – reported failure modes and error analysis; environmental factors affecting performance; computational constraints; generalization limitations; practical deployment barriers.

Studies were categorized across the four primary technical domains (species recognition, individual re-identification, soft biometrics, behavior/pose analysis), with cross-domain methods noted. Multiple independent reviewers coded a subset of papers to ensure consistency; disagreements were resolved through discussion.

#### Continental protected-species analysis: Dataset inclusion and exclusion criteria

For the continental protected-species analysis presented in Figure 3, two categories of dataset were excluded from the per-continent coding. First, general-purpose computer vision benchmarks spanning multiple continents without clear geographic attribution—specifically ImageNet and its derivatives (ImageNet-1k, ImageNet-21k)—were removed because their species composition is not tied to any single continent, rendering them unsuitable for a per-continent conservation analysis. Second, the Animal Kingdom benchmark,^56^ which intentionally covers 850 species across all continents as a broad computer-vision resource, was excluded because its protected-species counts for Africa (*n* = 50) and Asia (*n* = 40) are two to three times higher than any field-based study in the corpus, distorting continental distributions and summary statistics. The final corpus for Figure 3 comprises the 159 primary stxddudies with verifiable single-continent attribution.

### Quantification and statistical analysis

This review synthesizes published literature rather than conducting primary statistical analyses. Performance metrics are reported as presented in original studies. We did not conduct formal meta-analysis due to heterogeneous evaluation protocols, diverse datasets, and varying experimental conditions across studies preventing meaningful quantitative synthesis.

When comparing methods, we note differences in evaluation datasets, training data quantities, and experimental conditions that affect direct performance comparison. We emphasize relative improvements within studies using consistent methodology over cross-study absolute performance comparisons.

For assessing research trends, we quantified: (1) number of publications per year across technical domains, (2) architectural adoption rates (% of papers using CNNs vs. Transformers vs. hybrid approaches over time), (3) species coverage across datasets, and (4) geographic distribution of study locations. These descriptive statistics characterize field evolution without inferential statistical testing.
