# Supplementary material for: From pixels to conservation: Deep learning for automated monitoring of rare and endangered wildlife
Source: iScience. 2026 Sep 10;29(10):116669. doi: 10.1016/j.isci.2026.116669 (PMC13586580; doi:10.1016/j.isci.2026.116669)
Supplement: Supplementary file 1 [file mmc1.pdf]

iScience, Volume *29*

Supplemental information

From pixels to conservation: Deep learning for automated monitoring of rare and endangered wildlife

Donghua Xuan, Yuchen Cai, Minxi Xu, Chao Long, and Nian Cai

Supplemental Information

Document S1

This PDF contains Figures S1–S3 and Tables S1–S2 referenced in the main text.

Paper Title: *From Pixels to Conservation: Deep Learning for Automated Monitoring of Rare and Endangered Wildlife*

Authors:
Donghua Xuan, Yuchen Cai, Minxi Xu, Chao Long, and Nian Cai

Journal:
iScience

Figure S1. Dataset Scale and Purpose Distribution
(Related to Results – Datasets and Benchmarks for Wildlife Conservation)

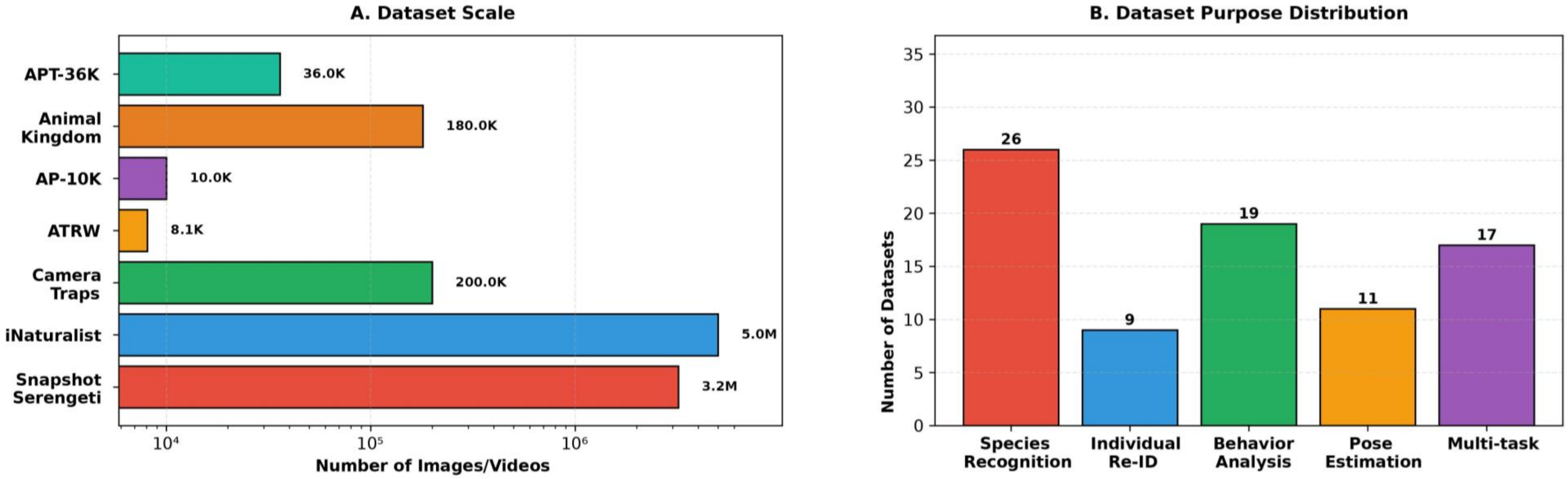

Figure S1. Dataset Scale and Purpose Distribution, related to Results – Datasets and Benchmarks for Wildlife Conservation.

(A) Dataset Scale. *Horizontal bar chart showing the total image or video count for seven representative publicly available wildlife monitoring datasets plotted on a* $\log_{10}$ *scale (x-axis). Each bar is colour-coded by dataset: iNaturalist (blue, 5.0M); Snapshot Serengeti (red, 3.2M); Camera Traps multi-site collection (green, 200.0K); Animal Kingdom (orange, 180.0K); APT-36K (teal, 36.0K); AP-10K (purple, 10.0K); ATRW (amber, 8.1K). Annotated values at bar ends indicate the exact sample count.*

(B) Dataset Purpose Distribution. *Bar chart showing the number of datasets (y-axis, 0–35) from the 35 publicly available datasets reviewed in Table S1 that support each of five task categories (x-axis). Bars show: Species Recognition (n = 26); Individual Re-ID (n = 9); Behavior Analysis (n = 19); Pose Estimation (n = 11); Multi-task (n = 17, datasets supporting ≥ 2 task categories simultaneously). Bar values are annotated above each bar. Task categories are not mutually exclusive; a single dataset may contribute to more than one bar.*

Figure S2. Performance Benchmarks Across Technical Domains
(Related to Results – Performance Benchmarks)

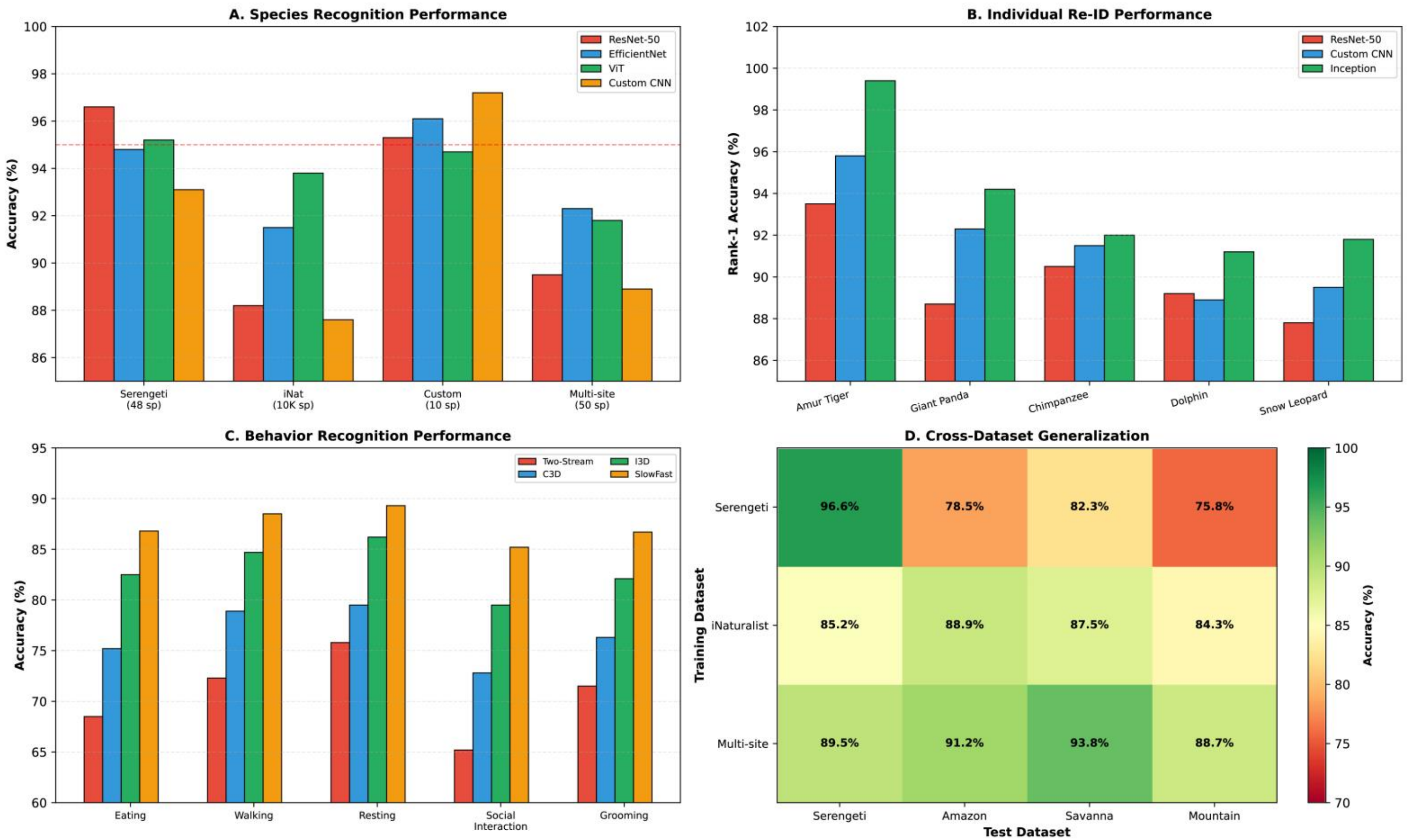

Figure S2. Performance Benchmarks Across Technical Domains, related to Results.

(A) Species Recognition Performance. *Grouped bar chart comparing Top-1 accuracy (%, x-axis; range 86–100%) across four benchmark datasets (y-axis: Serengeti, iNat, Custom, Multi-site) for four architecture types: ResNet-50, EfficientNet, ViT, and Custom CNN. Accuracy values are annotated within bars. The heatmap colour scale transitions from red (lower accuracy) through yellow to green (higher accuracy).*

(B) Individual Re-ID Performance. *Grouped bar chart showing Rank-1 accuracy (%, x-axis; range 86–102%) across five species (y-axis: Amur Tiger, Giant Panda, Chimpanzee, Dolphin, Snow Leopard) for three architecture types: ResNet-50, Custom CNN, and Inception. Rank-1 accuracy represents the proportion of queries where the correct individual ranks first in the retrieval list.*

(C) Behavior Recognition Performance. *Grouped bar chart comparing accuracy (%, x-axis; range 60–95%) across five behavior categories (y-axis: Eating, Walking, Resting, Social, Grooming) for four video architecture types: Two-Stream, C3D, I3D, and SlowFast.*

(D) Cross-Dataset Generalization. *Heatmap showing species recognition accuracy (%, annotated in each cell; range 75.8–96.6%) for a 4 × 3 matrix of training dataset (rows: Serengeti, iNaturalist, Multi-site) versus test dataset (columns: Serengeti, Amazon, Savanna, Mountain) combinations. Colour scale: green = higher accuracy, red = lower accuracy. Diagonal cells (same-domain) consistently achieve the highest accuracy (96.6%, 93.8%, 91.2%), while off-diagonal cells reveal cross-domain performance drops, illustrating dataset-specific generalisation limitations.*

Figure S3. Detection Architecture Performance Comparison

(Related to Results – Unified Detection-Classification Pipelines)

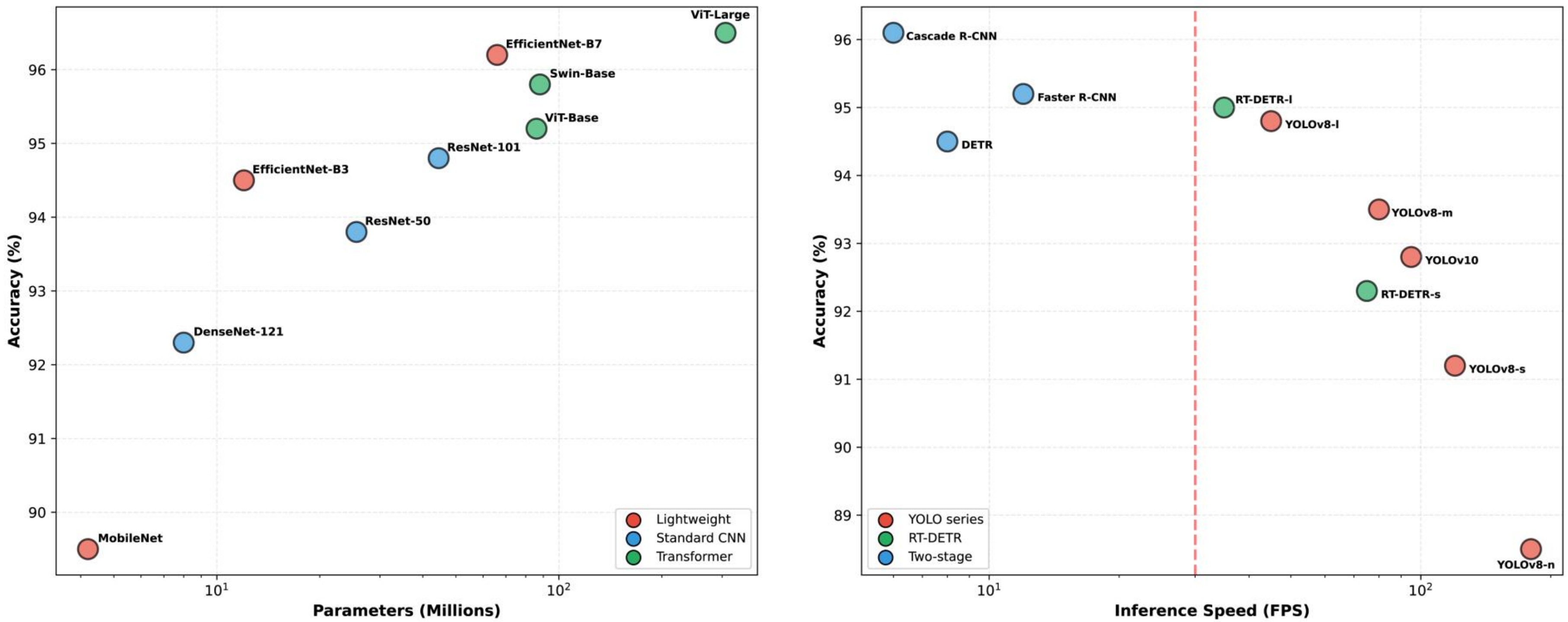

Figure S3. Detection Architecture Performance Comparison, related to Results – Unified Detection-Classification Pipelines.

(A) Accuracy vs. Model Size. *Scatter plot comparing Top-1 accuracy (%, y-axis; range 90–96%) versus model parameter count (Millions, x-axis; $\log_{10}$ scale: 10^1–10^2) for nine backbone architectures. Points are colour-coded by architecture family: Lightweight models (MobileNet); Standard CNN (ResNet-50, EfficientNet-B3, DenseNet-121, ResNet-101, EfficientNet-B7); Transformer (ViT-Base, Swin-Base, ViT-Large). Transformer architectures occupy the upper-right region, indicating*

higher accuracy at larger parameter counts, while lightweight models cluster at lower-left.

(B) Speed vs. Accuracy Trade-off. *Scatter plot comparing Top-1 accuracy (%, y-axis; range 89–96%) versus inference speed (FPS, x-axis;* log_{10} *scale:* 10^1*–*10^2*) for ten detection architectures. Points are colour-coded by detector family: YOLO series (YOLOv8-n, YOLOv8-s, YOLOv8-m, YOLOv8-l, YOLOv10); RT-DETR (RT-DETR-s, RT-DETR-l); Two-stage detectors (Faster R-CNN, DETR, Cascade R-CNN). YOLO-series models dominate the high-speed, moderate-accuracy region; two-stage detectors and RT-DETR achieve higher accuracy at lower FPS, reflecting their computational overhead. The figure supports the main-text discussion that YOLO variants are preferred for real-time field deployment, while two-stage detectors are suited for offline high-precision analysis.*

Table S1. Representative Datasets in Wildlife Monitoring Literature

(Related to Results – Datasets and Benchmarks for Wildlife Conservation)

Table S1.Comprehensive dataset summary

NO.	Dataset	Species	Quantity and Format	Feature	Task	Adress
1	Animal Kingdom [1]	850 species total (e.g., chimpanzees)	50 hours annotated video, 30K sequences, 33K frames	Sourced from YouTube; suitable for various tasks like animal behavior understanding	BA + PE + MT	link
2	Animal2–v1 [2]	Bear, deer, elephant, leopard, monkey, tiger, wild boar	9.9k images, uneven distribution per species	Wildlife dataset; average image quality	SR	link
3	AP-10K [3]	Lion, tiger, etc.	54 species, 10k images	Large-scale pose estimation dataset	PE	link
4	APT-36K [4]	Lion, tiger, etc.	30 animal species, 2.4k video clips, 36k frames	Single-frame, cross-species, pose estimation & tracking	PE + Tracking + MT	link
5	ATRW [5]	Wild Amur (Siberian) tiger	8k+ video clips, 92 individual tigers	Benchmark dataset for wild tiger re-identification; videos include diverse angles, poses, and behaviors	IR + PE + MT	link
6	BaboonLand Dataset [6]	Baboon	30k images, half-hour dense tracking, 20h behavior data	Long-tailed distribution, covers various environmental backgrounds	SR + BA + MT	link
7	Białowieża National Park (BNP) [7]	European forest wildlife (e.g., lynx, wolf, moose)	~60k images, typical quality, real camera trap data	From one of Europe's last primeval forests; includes rare species; high value for conservation research	SR	link
8	Caltech Camera Traps [7]	Bear, deer, raccoon, coyote, etc.	7.9k images, 46.5k animal instances	Camera trap dataset for studying animal population distribution, behavior patterns, etc.	SR	link
9	ChimpACT [8]	Chimpanzee	163 videos, ~160.5k frames	Rich annotations, tracking chimpanzee growth	IR + PE + BA + MT	link
10	ChimpBehave [8]	Chimpanzee	2+ hours video, 1.4k annotated clips	Expert annotated, imbalanced data	BA	link
11	ChimpFace [9]	Chimpanzee	5.6k images, 95 individuals	Individual identification, behavior study	IR	link
12	C-Tai [10]	Chimpanzee	78 chimps, 5.1k images, 5 age groups	Shot in wild natural habitat; diverse poses, expressions, behaviors	IR	link
13	C-Zoo [10]	Chimpanzee	24 chimps, 2.1k images, 4 age groups	Zoo setting; stable background and lighting	IR	link
14	Elephant Listening Project [11]	African Elephant	2.1k images, 276 individuals, 1M+ hours audio	Study habitat, population distribution, behavior patterns	SR + BA + MT	需邮件申请
15	ENA24 [12]	23 categories (e.g., American Crow, American Black Bear, Dog)	~10k camera trap images, various resolutions	Eastern North America field camera traps; reflects real wildlife status & behavior	SR	link
16	ENA24-detection [7]	European mammals (e.g., Brown Bear, Wolf, Roe Deer)	~10k camera trap images, bounding box annotations	Good data quality; clear images, detailed annotations	SR	link
17	HappyWhale [9]	Whale (Humpback)	4.3k individuals, 9.9k images	Individual whale identification; fluke images	IR	link
18	Idaho Camera Traps [7] Schneider	Moose, deer, etc.	~1.5M images, 40k pictures, 150k animal instances	~70.5% empty images; for analyzing animal activity under different environmental conditions	SR	link

19	iNaturalist [13]	Mammals, reptiles, amphibians, etc., 127k+ species	2.7M images, various resolutions	Highly imbalanced; images from various cameras worldwide	SR	link
20	iPanda-30 [14]	Panda	30 pandas, 3.6k images	High-quality dataset for identification & behavior analysis	IR + BA + MT	link
21	Kenyan Animal Behavior Recognition (KABR) [15]	Mammals, birds, reptiles, etc., 50 species	10k video clips, 7 animal behavior types	Good quality; covers diverse animal behaviors; from Kenyan nature reserves	BA	link
22	LoTE-Animal [16]	11 endangered species (e.g., Giant Panda, Red Panda, Golden Snub-nosed Monkey)	500K videos, 1.2M images, 10K video/action recognition tasks	Rich environmental variations (seasons, weather, etc.)	SR + PE + BA + MT	link
23	MammalClub [16]	24 mammals (e.g., Brown Bear, Cow, Leopard)	118.6k images; sub-datasets for species/ID/behavior recognition	Camera trap dataset for population distribution, behavior patterns, etc.	SR + IR + BA + MT	link
24	MammalNet[17]	173 mammal categories	18k videos (~539 hours), 20k trimmed instances	Large and diverse dataset for mammal identification & behavior understanding	SR + BA + MT	link
25	Snapshot USA [18]	White-tailed deer, raccoon, etc.	3.4M images, 308 species	Good quality; rich species info; observations across seasons/times	SR	link
26	Missouri Camera Traps [7]	Deer, raccoon, coyote, etc.	~105k images, varying quality (empty/occluded /blurry)	Real camera trap data; includes time, location, species labels; suitable for ecology & long-tail/imbalanced classification research	SR	link
27	MS COCO 2017 [19]	Elephant, bear, etc.	118.3k images, 80 annotated categories	High quality; clear images; detailed & accurate annotations	SR + PE + MT	link
28	NACTI [12]	28 animal categories (e.g., Cow, Pig, Deer)	~3.7M camera trap images, 5 locations	Large scale, species-rich; for training/evaluating biodiversity monitoring models	SR	link
29	North American Camera Traps [7]	Various North American wildlife	~2.7M images, varying quality, aggregated from multiple datasets	Large-scale, multi-location camera trap integration; aims to advance general wildlife monitoring models	SR	link
30	OpenApePose [20]	Great Apes	71.9k photos, 16 body keypoints	Pose estimation; multi-source data	PE	link
31	PanAf20 [21]	Wild Chimpanzee	500 video clips, 180k+ frames	Frame-level annotations; imbalanced action distribution	SR + BA + MT	link
32	Snapshot Serengeti [7]	Lions, cheetahs, leopards, elephants, etc.	7.1M camera trap images	Massive volume, species-rich, classic long-tailed distribution	SR	link
33	Tierschnappschuss [7]	Fox, deer, etc.	~115k annotated images, typical quality, real camera trap data	Provides species and count labels; for biodiversity monitoring	SR	link
34	WCS Camera Trap Data [7]	Tropical wildlife	1M+ images	High authenticity; includes timestamp & geolocation	SR	link
35	Wild Animal Facing Extinction [19]	Elephant, lion, cheetah, giraffe, etc.	7.6k images, manually annotated rhino images	Aims to mitigate wildlife poaching; semi-automatic annotation framework	SR + PE + MT	link

***Note:** This table summarises 35 publicly available datasets used in wildlife monitoring research, detailing dataset name, target taxa, data scale and format, salient features, and access information. Taxa covered include primates, large felids, ungulates, marine mammals, birds, and herpetofauna; data modalities range from camera-trap imagery and surveillance video to keypoint-annotated video and audio recordings. These datasets support tasks including species recognition, individual re-identification, behavioural analysis, pose estimation, and population monitoring. Most datasets*

are accessible via public links; some require direct application to data custodians (e.g., Elephant Listening Project). ***Abbreviations and symbols:*** *No., number; k/K, thousand; M, million; h, hours; ~, approximately; +, more than; CT, camera trap; spp., species; inst., instances; id, identity/identification; annot., annotations; behav., behaviour/behavior; vid, video clips; fr, frames; img, images; kp, keypoints; bbox, bounding box; SR, species recognition; ReID, individual re-identification; BA, behavioural analysis; PE, pose estimation; PM, population monitoring.*

Table_S2. Conservation impact case studies (Related to Introduction and Results)

Table S2.Conservation impact case studies

Species/Project	Location	Technology Used	Conservation Goal	Performance Achieved	Impact/Outcome	Time Period	Status
Giant Panda Monitoring	Wolong NR, China	ResNet-based ID + Age estimation	Population monitoring & demographics	97.2% ID accuracy, 1.5yr age error	Reduced manual analysis from 12 months to 2 weeks; Enabled real-time population tracking	2020-2026	Ongoing
Amur Tiger Anti-poaching	Russia-China border	Stripe-based Re-ID (99.37%)	Individual tracking & anti-poaching	>99% Re-ID accuracy	Identified 92 individuals; Tracked movement patterns; Supported anti-poaching patrols	2020-2026	Successful
Chimpanzee Social Networks	Gombe, Tanzania	Face recognition + behavior analysis	Community dynamics & health monitoring	>90% individual recognition	Mapped social networks; Identified behavioral changes; Disease outbreak detection	2018-2026	Long-term
Marine Mammal Tracking	Global oceans	Natural marking Re-ID	Migration patterns & population	Ocean-scale tracking	Multi-year individual tracking; Migration route mapping; Population estimates	2015-2026	Multi-year
Snow Leopard Assessment	Central Asia (5 countries)	Camera trap AI + Re-ID	Population assessment & habitat use	91.2% Re-ID accuracy	First comprehensive population estimate; Habitat connectivity analysis	2021-2025	Completed
African Elephant Census	Multiple African parks	Drone-based detection	Aerial population counting	95%+ detection rate	Large-area census in days vs. months; Real-time poaching threat detection	2022-2026	Ongoing

Penguin Colony Monitoring	Antarctica	Automated counting from drones	Climate impact assessment	98% counting accuracy	Annual colony size tracking; Climate change impact quantification	2019-2026	Long-term
Orangutan Behavior Study	Borneo, Indonesia	Pose estimation + behavior	Health & rehabilitation monitoring	88% behavior classification	Rehabilitation success tracking; Individual health monitoring	2020-2025	Ongoing
Sea Turtle Nesting	Multiple beaches	Automated nest detection	Nesting success & hatchling survival	94% nest detection	Real-time nest monitoring; Predator threat alerts; Hatchling survival rates	2018-2026	Successful
Jaguar Corridor Mapping	Central/South America	Camera trap networks + AI	Connectivity & corridor identification	93% species ID, movement tracking	Identified critical corridors; Informed land-use policy; Reduced habitat fragmentation	2019-2025	Ongoing

Note: Conservation impact case studies of AI-based species identification and monitoring. Projects are selected to illustrate diverse taxonomic groups (mammals, birds, reptiles), geographic regions, conservation objectives (e.g., population monitoring, anti-poaching, habitat connectivity), reported performance (accuracy/detection rates), and conservation outcomes (e.g., reduced manual effort, population estimates, policy impact). Time periods and status indicate project duration and progress.

REFERENCES:

1. Ng, X.L., Ong, K.E., Zheng, Q., Ni, Y., Yeo, S.Y., and Liu, J. (2022). Animal Kingdom: A Large and Diverse Dataset for Animal Behavior Understanding. In Proceedings of the IEEE/CVF conference on computer vision and pattern recognition, pp. 19023-19034.
2. Bhagabati, B., Sarma, K.K., and Bora, K.C. (2024). An automated approach for human-animal conflict minimisation in Assam and protection of wildlife around the Kaziranga National Park using YOLO and SENet Attention Framework. Ecological Informatics *79*, 102398. 10.1016/j.ecoinf.2023.102398.
3. Yu, H., Xu, Y., Zhang, J., Zhao, W., Guan, Z., and Tao, D. (2021). AP-10K: A Benchmark for Animal Pose Estimation in the Wild. In Neural Information Processing Systems Track on Datasets and Benchmarks 1 (NeurIPS Datasets and Benchmarks 2021), December 2021.
4. Yang, Y., Yang, J., Xu, Y., Zhang, J., Lan, L., and Tao, D. (2022). APT-36K: A Large-Scale Benchmark for Animal Pose Estimation and Tracking. Advances in Neural Information Processing Systems *35*, 17301-17313.
5. Li, S., Li, J., Tang, H., Qian, R., and Lin, W. (2020). ATRW: A benchmark for amur tiger re-identification in the wild. Proceedings of the 28th ACM International Conference on Multimedia (MM '20). Association for Computing Machinery.
6. Duporge, I., Kholiavchenko, M., Harel, R., Wolf, S., Rubenstein, D.I., Crofoot, M.C., Berger-Wolf, T., Lee, S.J., Barreau, J., Kline, J., et al. (2025). BaboonLand Dataset: Tracking Primates in the Wild and Automating Behaviour Recognition from Drone Videos. International Journal of Computer Vision *133*, 6578-6589. 10.1007/s11263-025-02493-5.
7. Schindler, F., and Steinhage, V. (2021). Identification of animals and recognition of their actions in wildlife videos using deep learning techniques. Ecological Informatics *61*, 101215.
8. Fuchs, M., Genty, E., Bangerter, A., Zuberbuhler, K., Odobez, J.M., and Cotofrei, P. (2025). From Forest to Zoo: Great Ape Behavior Recognition with ChimpBehave. Int J Comput Vis *133*, 6668-6688. 10.1007/s11263-025-02484-6.
9. Schneider, S., Greenberg, S., Taylor, G.W., and Kremer, S.C. (2020). Three critical factors affecting automated image species recognition performance for camera traps. Ecology and evolution *10*, 3503-3517. 10.1002/ece3.6147.
10. Freytag, A., Rodner, E., Simon, M., Loos, A., Kühl, H.S., and Denzler, J. (2016). Chimpanzee Faces in the Wild: Log-Euclidean CNNs for Predicting Identities and Attributes of Primates. In German Conference on Pattern Recognition, (Springer), pp. 51–63.
11. Körschens, M., and Denzler, J. (2019). ELPephants: A Fine-Grained Dataset for Elephant Re-Identification.
12. Sun, T., Liu, R., Wang, Z., Chen, Y., and Li, S. (2023). Basic behavior recognition of yaks based on improved SlowFast network. Ecological Informatics *78*, 102313. 10.1016/j.ecoinf.2023.102313.
13. Cardoso, A.S., Bryukhova, S., Renna, F., Reino, L., Xu, C., Xiao, Z., Correia, R., Di Minin, E., Ribeiro, J., and Vaz, A.S. (2023). Detecting wildlife trafficking in images from online platforms: A test case using deep learning with pangolin images. Biological Conservation *279*, 109905. 10.1016/j.biocon.2023.109905.
14. Ding, R., Wang, L., Zhang, Q., Niu, Z., Zheng, N., and Hua, G. (2020). Fine-Grained Giant Panda Identification. In ICASSP 2020-2020
15. Kholiavchenko, M., Kline, J., Ramirez, M., Stevens, S., Sheets, A., Babu, R., Banerji, N.,

Campolongo, E., Thompson, M., and Van Tiel, N. (2024). KABR: In-Situ Dataset for Kenyan Animal Behavior Recognition from Drone Videos. In Proceedings of the IEEE/CVF winter conference on applications of computer vision, pp. 31-40.

16. Lu, W., Zhao, Y., Wang, J., Zheng, Z., Feng, L., and Tang, J. (2023). MammalClub: An Annotated Wild Mammal Dataset for Species Recognition, Individual Identification, and Behavior Recognition. Electronics *12*, 4506. 10.3390/electronics12214506.
17. Chen, J., Hu, M., Coker, D.J., Berumen, M.L., Costelloe, B., Beery, S., Rohrbach, A., and Elhoseiny, M. (2023). MammalNet: A Large-Scale Video Benchmark for Mammal Recognition and Behavior Understanding. In Proceedings of the IEEE/CVF conference on computer vision and pattern recognition, pp. 13052-13061.
18. Herrera, D.J., Schalk, C.M., Jensen, A.J., Goldstein, B.R., Rooney, B.R., Kays, R., McShea, W.J., and Cove, M.V. (2025). iNaturalist and structured mammal surveys reflect similar species richness but capture different species pools across the United States. Ecology and Evolution *15*, e71805.
19. Sapkota, R., Flores-Calero, M., Qureshi, R., Badgujar, C., Nepal, U., Poulose, A., Zeno, P., Vaddevolu, U.B.P., Khan, S., and Shoman, M. (2025). YOLO advances to its genesis: A decadal and comprehensive review of the You Only Look Once (YOLO) series. Artificial Intelligence Review *58*, 274. 10.1007/s10462-025-11253-3.
20. Desai, N., Bala, P., Richardson, R., Raper, J., Zimmermann, J., and Hayden, B. (2023). OpenApePose, a database of annotated ape photographs for pose estimation. elife *12*, RP86873.
21. Brookes, O., Mirmehdi, M., Stephens, C., Angedakin, S., Corogenes, K., Dowd, D., Dieguez, P., Hicks, T.C., Jones, S., and Lee, K. (2024). PanAf20K: A Large Video Dataset for Wild Ape Detection and Behaviour Recognition. International Journal of Computer Vision *132*, 3086-3102.
